# Advanced Microbial Taxonomy Combined with Genome-Based-Approaches Reveals that *Vibrio astriarenae* sp. nov., an Agarolytic Marine Bacterium, Forms a New Clade in *Vibrionaceae*

**DOI:** 10.1371/journal.pone.0136279

**Published:** 2015-08-27

**Authors:** Nurhidayu Al-saari, Feng Gao, Amin A.K.M. Rohul, Kazumichi Sato, Keisuke Sato, Sayaka Mino, Wataru Suda, Kenshiro Oshima, Masahira Hattori, Moriya Ohkuma, Pedro M. Meirelles, Fabiano L. Thompson, Cristiane Thompson, Gilberto M. A. Filho, Bruno Gomez-Gil, Toko Sawabe, Tomoo Sawabe

**Affiliations:** 1 Laboratory of Microbiology, Faculty of Fisheries, Hokkaido University, Minato-cho, Hakodate, Japan; 2 Laboratory of Metagenomics, Graduate School of Frontier Sciences, University of Tokyo, Kashiwa, Japan; 3 Department of Microbiology and Immunology, Keio University School of Medicine, Tokyo, Japan; 4 Graduate School of Advanced Science and Engineering, Waseda University, Tokyo, Japan; 5 Microbe Division/Japan Collection of Microorganisms, RIKEN BioResource Center, Ibaraki, Japan; 6 Institute of Biology, SAGE-COPPE, Federal University of Rio de Janeiro (UFRJ), Rio de Janeiro, Brazil; 7 Rio de Janeiro Botanical Garden, Rio de Janeiro, Brazil; 8 CIAD, AC Mazatlan Unit for Aquaculture and Environmental Management, Mazatlán, México; 9 Department of Food and Nutrition, Hakodate Junior College, Hakodate, Japan; The University of Hong Kong, HONG KONG

## Abstract

Advances in genomic microbial taxonomy have opened the way to create a more universal and transparent concept of species but is still in a transitional stage towards becoming a defining robust criteria for describing new microbial species with minimum features obtained using both genome and classical polyphasic taxonomies. Here we performed advanced microbial taxonomies combined with both genome-based and classical approaches for new agarolytic vibrio isolates to describe not only a novel *Vibrio* species but also a member of a new *Vibrio* clade. Two novel vibrio strains (*Vibrio astriarenae* sp. nov. C7^T^ and C20) showing agarolytic, halophilic and fermentative metabolic activity were isolated from a seawater sample collected in a coral reef in Okinawa. Intraspecific similarities of the isolates were identical in both sequences on the 16S rRNA and *pyrH* genes, but the closest relatives on the molecular phylogenetic trees on the basis of 16S rRNA and *pyrH* gene sequences were *V*. *hangzhouensis* JCM 15146^T^ (97.8% similarity) and *V*. *agarivorans* CECT 5085^T^ (97.3% similarity), respectively. Further multilocus sequence analysis (MLSA) on the basis of 8 protein coding genes (*ftsZ*, *gapA*, *gyrB*, *mreB*, *pyrH*, *recA*, *rpoA*, and *topA*) obtained by the genome sequences clearly showed the *V*. *astriarenae* strain C7^T^ and C20 formed a distinct new clade protruded next to *V*. *agarivorans* CECT 5085^T^. The singleton *V*. *agarivorans* has never been included in previous MLSA of *Vibrionaceae* due to the lack of some gene sequences. Now the gene sequences are completed and analysis of 100 taxa in total provided a clear picture describing the association of *V*. *agarivorans* into pre-existing concatenated network tree and concluded its relationship to our vibrio strains. Experimental DNA-DNA hybridization (DDH) data showed that the strains C7^T^ and C20 were conspecific but were separated from all of the other *Vibrio* species related on the basis of both 16S rRNA and *pyrH* gene phylogenies (e.g., *V*. *agarivorans* CECT 5085^T^, *V*. *hangzhouensis* JCM 15146^T^ *V*. *maritimus* LMG 25439^T^, and *V*. *variabilis* LMG 25438^T^). *In silico* DDH data also supported the genomic relationship. The strains C7^T^ also had less than 95% average amino acid identity (AAI) and average nucleotide identity (ANI) towards *V*. *maritimus* C210, *V*. *variabilis* C206, and *V*. *mediterranei* AK1^T^, *V*. *brasiliensis* LMG 20546^T^, *V*. *orientalis* ATCC 33934^T^, and *V*. *sinaloensis* DSM 21326. The name *Vibrio astriarenae* sp. nov. is proposed with C7 as the type strains. Both *V*. *agarivorans* CECT 5058^T^ and *V*. *astriarenae* C7^T^ are members of the newest clade of *Vibrionaceae* named Agarivorans.

## Introduction

Advances in genomic microbial taxonomy have opened the way to create a more universal and transparent concept of species but is still in a transitional stage towards becoming a defining robust criteria for describing new microbial species with minimum features obtained using both genome and classical polyphasic taxonomies [1–3]. Polyphasic taxonomy significantly contributed to bacterial description in the past by integrating the analysis of the phenotypic, genotypic (including chemotaxonomic) and phylogenetic characters of the isolates. It is predicted that the practice will soon be replaced with genomic microbial taxonomy in which the principles and practices are being developed. A lot of recent literature [2–6] have discussed these changes and are predicting its future impact on current microbial description and microbial taxonomy as a whole. This paper intended to implement the ideas proposed in the aforementioned literature to describe a new vibrio species and also to elaborate such matters as the practical usage and delineation of minimum features in microbial genome taxonomy. We demonstrate the actual transitional stage—an interphase in the evolution from polyphasic taxonomy to genomic microbial taxonomy and the need to describe the bacteria as comprehensively as possible.

We suggest that regardless of all the discrepancies, it is generally acknowledged that a universal species definition might somewhat resolve most of the problems in one way or another. Thus, in this paper, we attempt to describe a vibrio species using a recent proposed species definition by Rosselló-Móra and Amann [6] i.e. “*a category that circumscribes monophyletic*, *and genomically and phenotypically coherent populations of individuals that can be clearly discriminated from other such entities by means of standardized parameters*”. The parameters used permit an accurate classification of species via three major premises namely i) monophyly—demonstrating all members of the taxon in which a new species belongs to share a common evolutionary history using phylogenetic inferences; ii) genomic coherence—modulating the circumscription of the unit using a specific pre-determined threshold value executed using its respective method which corresponds to the observable phenotypes used for identification purposes, and iii) phenotypic coherence—organisms in the same taxon should display similar physiological, structural and ecological properties either through direct determination of their characteristics or prediction of the genome sequences [6]. All of the data could be obtained by whole genome sequences but we still need validations in the experiments, in particular the description of new bacterial lineages to increase the reliability of the genome-based taxonomic approach.

The genus *Vibrio* was proposed in 1854 for Gram-negative fermentative halophilic bacteria [7]. Today, a total of 110 species with valid nomenclature [8] in the genus *Vibrio* have been described. These bacteria are ubiquitous, highly heterogeneous [9] and the species evolution developed by both lateral (horizontal) and vertical gene transfer [10]. Thus, a thorough description is a prerequisite for describing a new species in the *Vibrionaceae* family. *Vibrio* species significantly contribute to the nutrient cycle by mediating organic matter decomposition. They have versatile metabolisms and are capable of degrading and fermenting complex organic matters such as polysaccharides [11]. Though the ability to consume agar, a complex polysaccharide composed of agarose and agaropectin, is known to be common among marine bacteria [12–13], it is not prevalent within the *Vibrio* species. Currently, *V*. *agarivorans* is the only agarolytic *Vibrio* that has been described thoroughly [12]. The species was isolated from Mediterranean seawater and reported to be able to degrade agar on both marine agar plates and thiosulphate-citrate-bile-sucrose (TCBS) agar [12]. In addition to some widely known agarases applications in food, cosmetic and medical industries, Chen et al. [14] and Dipakkore et al. [15] have demonstrated the efficient degradation of the cell walls of marine red algae in which cell wall was composed of agar. Thus, marine-derived agarases are valuable enzymes applicable for both cell biology and biotechnology in red algae. They are currently of growing interest due to their potential uses in the bioconversion of marine algal polysaccharides into energy feedstocks in biofuel industries [16]. Vibrios in particular, share such potential and our laboratory had successfully produced hydrogen from powdered brown macroalgae, *Sacchararina sculpera* [17]. Hence, further bioprospecting and the genomic survey of marine agarolytic bacteria will be greatly advantageous to the biofuel industry and at present is in high demand.

A two years survey of vibrios from the coral reef in Iriomote-Ishigaki National Park, Okinawa, Japan, obtained two agarolytic isolates with typical characteristics of *Vibrio* species. The isolates produced unpigmented colonies and displayed a shallow pit with pronounced diameter on ZoBell 2216E agar medium after 24 h of incubation at 30°C. During the primary vibrio survey, *pyrH* gene sequence analysis has placed these isolates into a distinct group not affiliated with any known *Vibrio* species. Thorough genomic and polyphasic taxonomies have strengthened the initial findings and further differentiate the isolates into their own unique group forming a distinct clade on basis of MLSA. We therefore concluded that these two bacteria may belong to a new species within the genus *Vibrio*. However, a close relationship of these isolates with *V*. *agarivorans* in terms of *pyrH* phylogeny and some phenotypic characters including the agarolytic activity demands a comprehensive MLSA. Hence, inclusion of completed eight housekeeping genes belongs to *V*. *agarivorans* CECT 5085^T^ into the pre-existing MLSA datasets were performed and reveals that the isolates shared the same clade with *V*. *agarivorans*. Both C7 and C20 are facultative anaerobes rods with polar flagella and capable of growth at 20–40°C at an optimum temperature of 30°C. This study provides the evidence and a detailed description of a novel agarolytic species for whom we propose the name *V*. *astriarenae* sp. nov. and falls into a novel clade named Agarivorans.

Here we performed advanced microbial taxonomy combining both genome-based and classical approaches for vibrio isolates belonging to a probable new clade species with the aim of describing a novel *Vibrio* species. Special emphasis is given to advantages and disadvantages of genome taxonomy.

## Materials and Methods

### Water sampling and bacterial strains

The study did not involve endangered or protected species. Two isolates of *V*. *astriarenae* sp. nov., strain C7^T^ and C20 were isolated from water samples collected from the coral reef in Iriomote-Ishigaki National Park, Okinawa, Japan. Specifically, the seawater samples were collected from the vicinity of Taketomi Island (24°20.5260' N; 124°05.6443' E) by SCUBA diving using underwater pumps. No specific permissions are required for water sampling activities in this location. The samples were then brought back to the lab and bacterial isolation was performed using thiosulphate-citrate-bile-sucrose (TCBS) medium (Nissui Pharmacy, Tokyo, Japan). Following incubation at 25°C for 24 h the isolates were then purified on ZoBell 2216E agar medium and incubated at 30°C. The strains were stored at -80°C using 20% glycerol-supplemented broth.

### Determination of moles percent G+C content and DNA-DNA hybridizations

Strains used in DNA-DNA hybridization experiment were C7^T^, C20, *V*. *maritimus* LMG 25439^T^, *V*. *variabilis* LMG 25438^T^, *V*. *brasiliensis* LMG 20546^T^, *V*. *agarivorans* CECT 5085^T^ and *V*. *hangzhouensis* JCM 15146^T^. Genomic DNAs of *V*. *astriarenae* C7^T^, *V*. *agarivorans* CECT 5085^T^, *V*. *brasiliensis* LMG 20546^T^ and *V*. *hangzhouensis* JCM 15146^T^ were used as probes. DNAs of bacterial strains were prepared according to the procedures of Marmur [18], with minor modifications. Moles percent G+C contents of DNA from *V*. *astriarenae* sp. nov., strain C7^T^ and C20 were determined using high-performance liquid chromatography (HPLC) [19]. DNA-DNA hybridization experiments were performed in microdilution wells using a fluorometric direct binding method [20]. DNAs of *V*. *astriarenae* sp. nov., strain C7^T^, *V*. *agarivorans*, *V*. *brasiliensis* and *V*. *hangzhouensis* were labeled with photobiotin (Vector Laboratories, Inc., Burlingame, CA). Four micro-grams of unlabeled single-stranded DNA were immobilized in microdilution wells (Immuron 200, FIA/LIA plate, black type, Greiner labotechnik, Germany), then a hybridization mixture containing 20 ng of labeled DNA was added to each microdilution well and the hybridization was performed under optimal conditions following pre-hybridization. Formamide concentration in the hybridization mixture was determined according to Meinkoth-Wahl [21]. The hybridization of the biotinylated DNA to immobilized DNAs was performed under optimal conditions (fixation at 37°C) following hybridization at 45°C and detected by fluorometry after binding streptavidin-β-galactosidase to labeled DNA. 4-Methylumbelliferyl-β-d-galactopyranoside (6 x 10^−4^ M; Wako, Osaka, Japan) was added to each well as fluorogenic substrate for β-galactosidase and incubated at 30°C. Then, the fluorescence intensity of each well was measured using a microplate reader (Infinite F200, Tecan, Switzerland) at wave length of 360 nm for excitation and 450 nm for emission. DNA-DNA homology was calculated according to Ezaki et al. [22].

### DNA amplification and sequencing

Bacterial DNAs of *V*. *astriarenae* sp. nov. strain C7^T^ and C20 for PCR were prepared using the Promega Wizard Genomic DNA extraction system according to the protocol provided by the manufacturer (Promega, Madison, WI). 100 ng of DNA template was used in a PCR to amplify the small-subunit rRNAs gene sequences. The initial denaturation step consisted of heating the reaction mixture at 94°C for 180 s, and this was followed by an annealing step (55°C for 60 s) and an extension step (72°C for 90 s). The thermal profile then consisted of 30 cycles of annealing at 55°C for 60 s, extension at 72°C for 90 s, and denaturation at 94°C for 60 s. The PCR products were analyzed on a 1.5% agarose gel with a molecular weight standard for quantification of the PCR yield. The amplification primers (24F and 1509R) used in this study gave a 1.5 kb long PCR product and corresponded to positions 25 to 1521 in the *E*. *coli* sequence. The PCR products producing a single band on agarose gels were purified using Promega Gel and PCR purification system (Promega, Madison, WI). Approximately 100 ng of template was directly sequenced using a BigDye terminator sequencing kit version 3.1 (Life Technologies, Carlsbad, CA) according to the protocol recommended by the manufacturer. DNA sequencing was performed using an Applied Biosystems model 3130x automated sequencer. Six DNA primers (24F, 800F, 1100F, 520R, 920R, 1509R) were used in the sequencing reactions [23].

### Phylogenetic analysis

**Fig 1:**
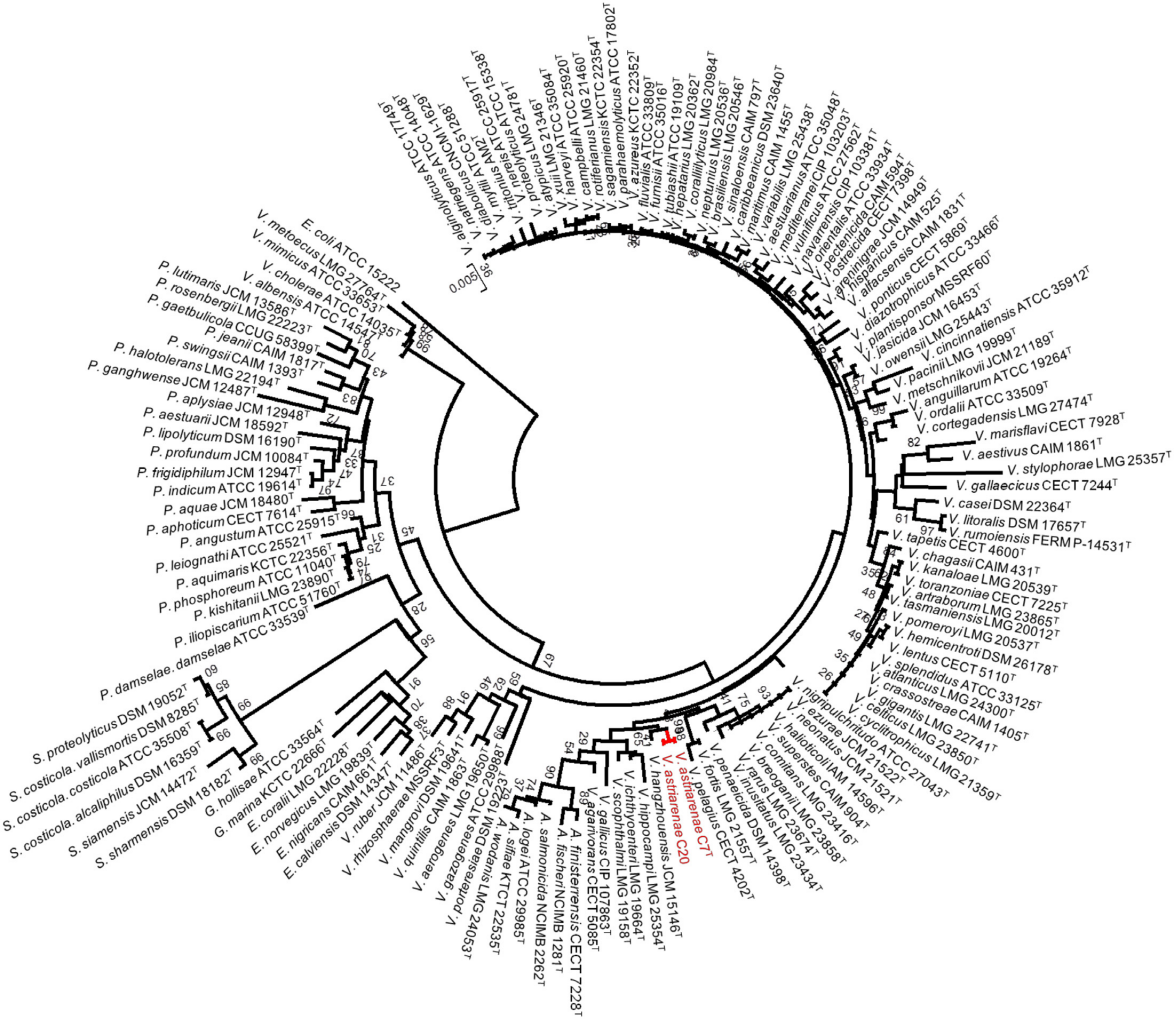
Phylogenetic tree on the basis of 16S rRNA gene sequences by neighbor-joining method. Bootstrap values are on 500 replicates. The topology of branch with the bootstrap value was also supported by maximum likelihood and maximum parsimony methods.

**Fig 2:**
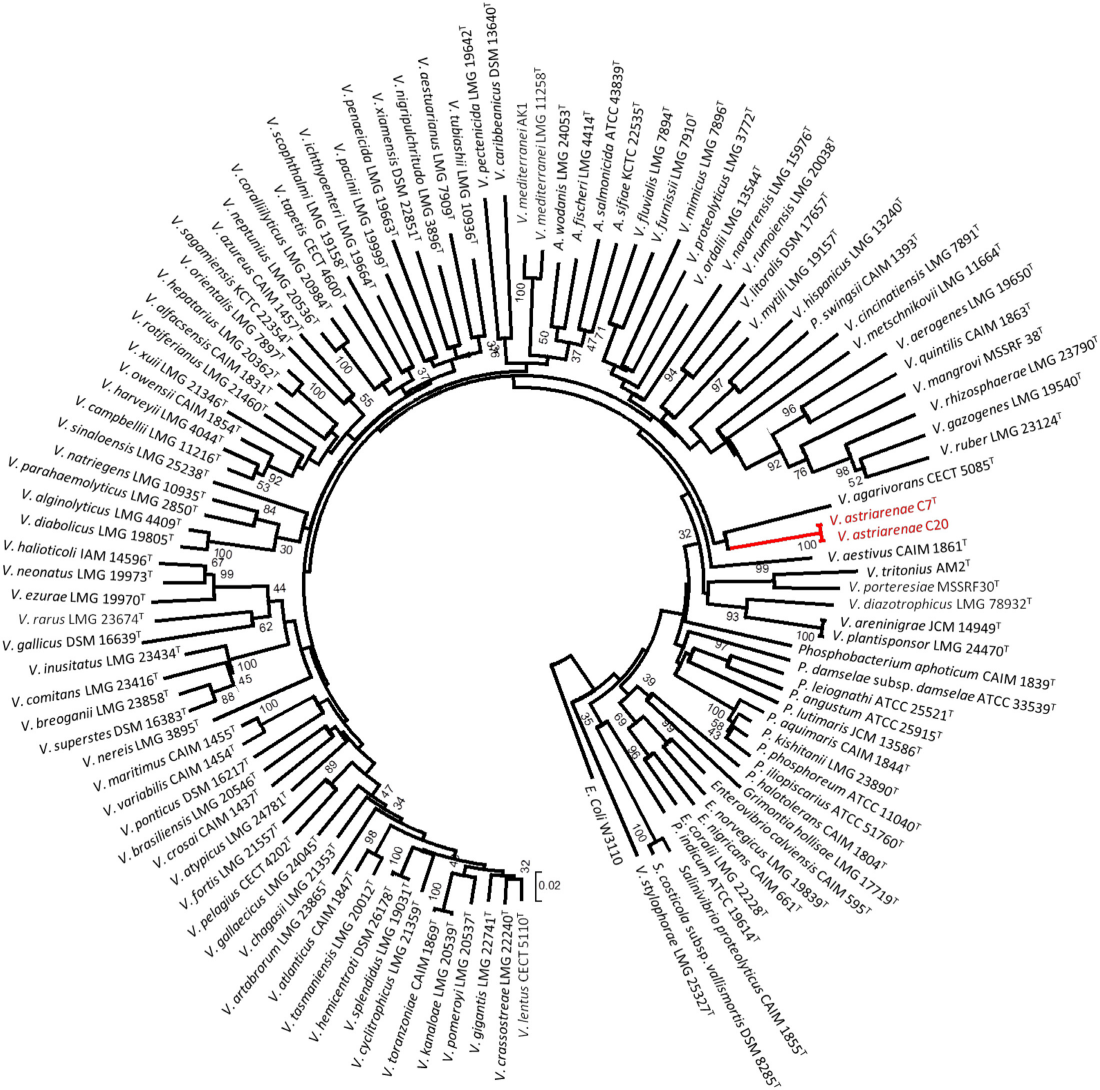
Phylogenetic tree on the basis of *pyrH* gene sequences by neighbor-joining method. The tree were drawn by MEGA and the gene sequences were corresponded to *E*. *coli* sequence at position 150 to 544. Bootstrap values are on 500 replicates. The topology of branch with the bootstrap value was also supported by maximum likelihood and maximum parsimony methods.

The sequences were aligned and studied using Clustal X version 2.1 [24] and MEGA programs version 6.06 [25]. The alignment was checked using the naked eye and corrected manually. In all phylogenetic analyses, we used the sequences determined in this study and small-subunit rRNA gene sequences obtained from the GenBank/EMBL/DDBJ databank. For Fig 1, the analysis was performed by applying the neighbor-joining method [26] with bootstrap values of 500 replicates to the full dataset of 141 sequences which includes 138 small-subunit rRNA gene sequences of type *Vibrionaceae* (S1 Table), two novel strains and *Escherichia coli* K-12. Evolutionary distances were computed using the Jukes-Cantor method. The robustness of topology was also checked using maximum parsimony and maximum likelihood methods with *E*. *coli* K-12 as the outgroup. The analyses were performed on the small-subunit rRNA gene sequences that corresponded to *E*. *coli* sequence at position 194 to 1403 and trees were drawn using the MEGA. Using a similar procedure, we also computed a phylogenetic analysis on the basis of *pyrH* gene sequences (Fig 2) of 113 *Vibrionaceae* species obtained from the public database.

### Genome sequencing and *in silico* DNA-DNA relatedness calculation

Draft genome sequences of strain C7^T^ were obtained using IonPGM sequencer with 28 redundancy. The genome sequences were assembled using Newbler ver. 2.8 into 336 contigs with 99142 of *N*_*50*_. The sequence was deposited in the DDBJ/GenBank/EMBL public database under accession numbers described below.

General genome features were determined through Rapid Annotations Using Subsystems Technology (The RAST server version 4.0) [27]. *In silico* DDH values of *V*. *astriarenae* sp. nov. C7^T^ were estimated against *V*. *brasiliensis* LMG 20546^T^, *V*. *maritimus* C210, *V*. *mediterranei* AK1, *V*. *variabilis* C206, *V*. *orientalis* ATCC 33934^T^, *V*. *sinaloensis* DSM 21326 and *V*. *tubiashii* ATCC 19109^T^ using Genome-to-Genome Distance Calculator (GGDC 2.0) [28]. This online tool infers genome-to-genome distances between pairs of entirely or partially sequenced genomes. Intergenomic distances were employed for wet-lab DDH prediction. Briefly, genome pairs were aligned with BLAST+ [29] to generate a set of high-scoring segment pairs (HSPs). The information they contained (e.g., the total number of identical base pairs) was transformed into a distance value by the best-fit formula, according to [28]. DDH prediction from intergenomic distance, including confidence intervals, was provided by a tested generalized linear model (GLM), [30] with log transformation [28]. Amino Acid Identity (AAI) and Average Nucleotide Identity (ANI) were calculated according to Konstantinidis and Tiedje [31] and Thompson et al. [32] respectively, on the same strains used for *in silico* DDH.

### Multilocus sequence analysis (MLSA)

**Fig 3:**
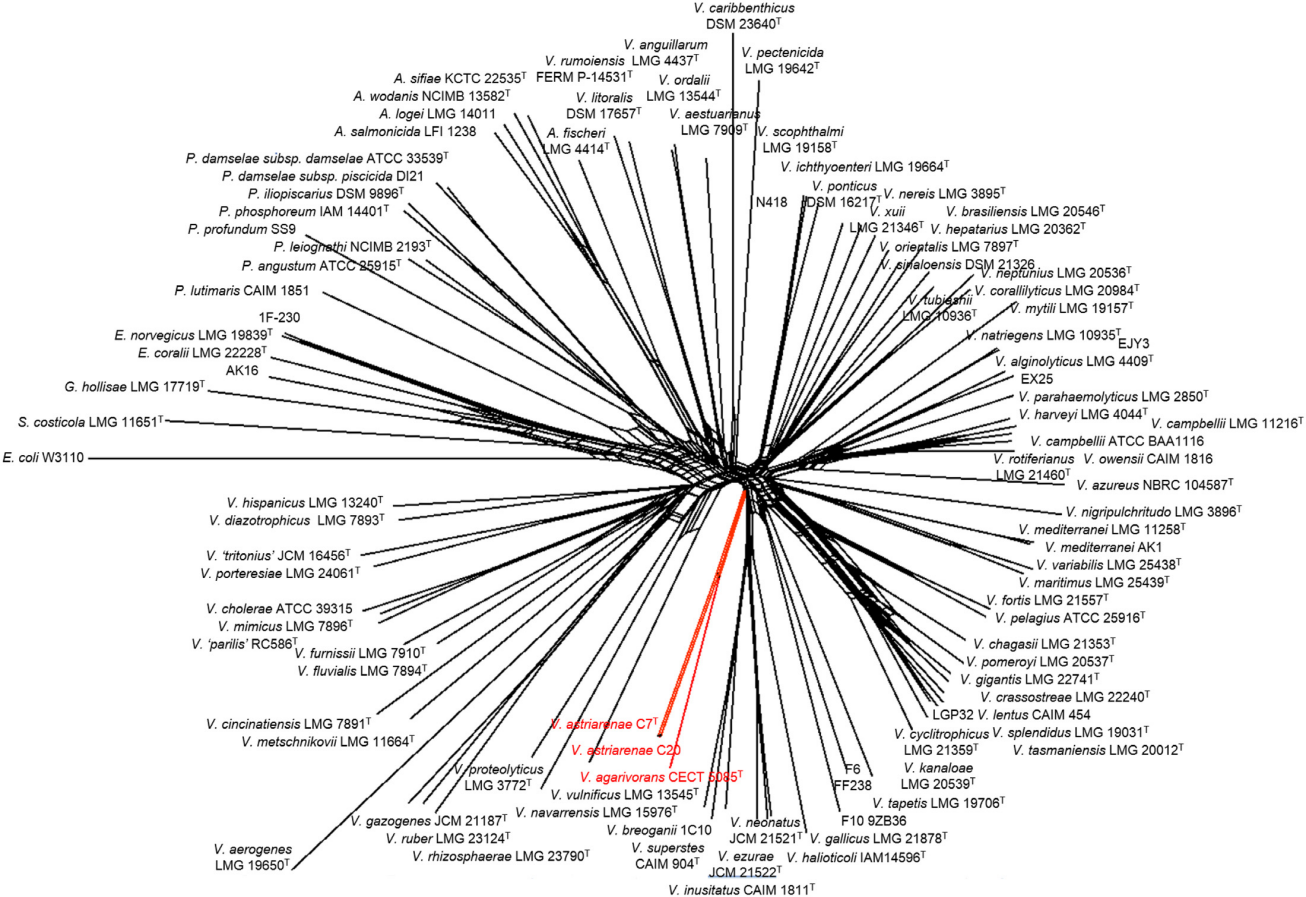
Concatenated split network tree based on eight gene loci. The *gapA*, *gyrB*, *ftsZ*, *mreB*, *pyrH*, *recA*, *rpoA*, and *topA* gene sequences of 100 taxa were concatenated including the representative of novel vibrios in the current study (*Vibrio astriarenae* sp. nov. C7^T^ and C20). Phylogenetic tree was generated using the SplitsTree4 program. *Vibrio astriarenae* sp. nov. C7^T^ and C20 formed a clade not associated any vibrio clades proposed previously.

Sequences of eight housekeeping genes (*ftsZ*, *gapA*, *gyrB*, *mreB*, *pyrH*, *recA*, *rpoA*, and *topA*) were retrieved from the genome sequence and used to infer the clade of *V*. *astriarenae* sp. nov., strain C7^T^ and C20 following a method described in Sawabe et al. [1,33]. Briefly, the sequences were aligned using the ClustalX 2.1 [24]. The domains used to construct the phylogenetic tree shown in Fig 3 were regions of the *ftsZ*, *gapA*, *gyrB*, *mreB*, *pyrH*, *recA*, *rpoA*, and *topA* genes of *Vibrionaceae*: positions 195–630, 225–861, 441–1026, 390–897, 171–543, 429–915, 87–873, and 570–990 (*V*. *cholera* O1 Eltor N16961 (AE003852) numbering), respectively. All regions investigated are in accordance with those used in earlier studies [1,33]. MEGA version 6 [25] was used to deduce the sequence similarity and the number of nucleotide and amino acid mutations. Another five housekeeping genes (*ftsZ*, *mreB*, *recA*, *rpoA*, and *topA*) of *V*. *agarivorans* CECT 5085^T^ were also retrieved in the same manner for a complete 100 taxa MLSA.

Split Decomposition Analysis (SDA) was performed according to Sawabe et al. [33] using SplitsTree version 4.13.1 [34], with a neighbour net drawing and Jukes-Cantor correction [1, 35]. The program was then used to generate the concatenated sequences of the eight housekeeping genes which served as basis for a phylogenetic analysis combined with NJ, MP, and ML analyses [23].

### Phenotypic characterization and genome based phenotypes

All strains were cultured on ZoBell 2216E agar medium [36] and their main characteristics were determined as described previously [37–42]. Phenotypic features were obtained directly from the whole genome sequences by automatically searching and database comparisons of the genes that define the metabolic pathways of each diagnostic feature and their regulatory genes as described previously [5]. Briefly, genes coding the proteins responsible for each feature were detected using the RAST program and the KEGG metabolic database following subsequent identification using BLASTP algorithm [5].

### Nucleotide sequence accession number

The genome data has been deposited at DDBJ/EMBL/GenBank under the accession numbers BBMQ01000001-336, BBMT01000001-45 and BBMS01000001-208 for *Vibrio astriarenae* sp. nov. C7^T^ (JCM 19233), *V*. *maritimus* C210 (JCM 19240) and *V*. *variabilis* C206 (JCM 19239), respectively.

The 16S rRNA gene sequences of C7^T^ and C20 performed by Sanger sequencing were deposited to GenBank under KP342514 and KP713778, respectively. Also, the housekeeping gene sequences for C20 and *V*. *agarivorans* CECT 5085^T^ performed by the same method were deposited under the accession number shown in S4 Table.

## Results and Discussion

### Monophyletic premise guarantees common evolutionary lineages

Tindall et al. [43] in their article have listed the key elements and set out a guide on how a prokaryote should be characterized for taxonomic purposes. The essence of the article is that the characterization of any new taxon should be as comprehensively as possible to place them within the existing hierarchical framework (Bacteriological Code, 1990 revision) and to provide a description of the taxa. In the current article, we described two potential new strains using both polyphasic and genomic approaches. Initially, the 16S rRNA gene sequences of strains C7^T^ and C20 were aligned by comparison to a database containing 138 aligned small-subunit rRNA gene sequences of type *Vibrionaceae*. The analyses by three different methods (NJ, ML and MP) showed no incongruence that they be included in the genus *Vibrio*; more precisely in a subgroup close to *V*. *hangzhouensis* JCM 15146^T^ (97.8% similarity) (Fig 1). The nucleotide sequences of the 16S rRNA gene of two strains of *V*. *astriarenae* sp. nov. were identical. Fig 1 depicts the node of final phylogenetic tree (shown in red) obtained using the NJ method with high bootstrap value (98%) and supported by ML and MP analyses. The sequence similarities to type strains of *Vibrionaceae* have further distinguished the novel strains from *V*. *agarivorans* by 97.3%. Furthermore, the similarities analyses to *V*. *maritimus* LMG 25439^T^, *V*. *variabilis* LMG 25438^T^ and *V*. *brasiliensis* LMG 20546^T^ were 98.8 to 99.0% which are in accordance to species delineation as proposed by Kita-Tsukamoto et al. [44]. The authors have drawn the circumscription border of new species in *Vibrionaceae* at ≤ 99.3% 16S rRNA similarity. On the *pyrH* gene phylogeny, *V*. *astriarenae* sp. nov. C7^T^ and C20 formed a robust clade with *V*. *agarivorans* CECT 5085^T^ as the closest related species (Fig 2).

Note that separate 16S rRNA and *pyrH* genes phylogenies of *V*. *astriarenae* sp. nov. C7^T^ and C20 are insufficient to demonstrate a monophyletic clade adjacent to the same reference type strain (close to *V*. *hangzhouensis* in 16S rRNA gene phylogeny, and to *V*. *agarivorans* based on *pyrH* gene) on its own. This is supported by previous researches [33,45] which showed the individual gene analyses are known to have low interspecies resolution in *Vibrionaceae*. The incongruence of the topologies on the basis of both genes may also indicate that C7^T^ and C20 probably form a novel clade. The MLSA of the eight housekeeping genes (Fig 3) provided a more robust inference of the evolutionary history of vibrios [33] verifying the monophyly of new candidates [6]. Previously [1], the singleton *V*. *agarivorans* was not included in eight genes analysis due to the lack of some gene sequences. The fact that both novel strains and *V*. *agarivorans* are sharing some phenotypic characters (described below) and their close relationship in *pyrH* gene phylogeny (Fig 2) demands a complete eight genes of *V*. *agarivorans* to be included to our multilocus sequences dataset. Thus, inclusion of multilocus sequences of *V*. *agarivorans* CECT 5085^T^ and *V*. *astriarenae* sp. nov., strain C7^T^ and C20 into the pre-existing clades data set [1] formed a branch that is strongly presumed to represent a new clade unrelated to any others (Fig 3). Sawabe et al. [1] has also described the other clades in great detail. It is noted that the species within each clade shared >20% DDH, <5% G+C (mol%), >85% MLSA sequence similarity and >89% AAI [1,33].

The concatenated network tree of 100 taxa of *Vibrionaceae* based on eight gene loci has positioned the strain C7 and C20 closely to *V*. *agarivorans* CECT 5085^T^ supported our earlier *pyrH*gene phylogeny. Simultaneously, the result is partly contradicted to the 16S rRNA-based phylogeny presented in Fig 1. The huge discrepant between 16S rRNA-based phylogeny and MLSA was also observed in the species description of *V*. *porteresiae* and *V*. *tritonius* AM2^T^ [1]. Similarly, the concatenated network tree based on eight genes revealed that both strains are sharing the same vibrio clade, Porteresiae as opposed to 16S rRNA gene sequence phylogeny which had initially placed *V*. *tritonius* closely to *V*. *furnissi* and *V*. *fluvialis* at 98% sequence similarity [1]. They also discovered similar incongruence in Mediterranei and Pectenicida clades. Single gene analysis is known to have different resolution according to the taxonomic groups due to different molecular clocks of the different genes [46]. Thompson et al. [46] in general suggested that the taxonomic resolution of 16S rRNA was restricted to genera differentiation among *Vibrionaceae* rather than the differentiation of species. This suggestion may partly explain the discrepancies. However, above all, the analyses presented herein have strongly proved the novelty of *V*. *astriarenae* sp. nov. C7^T^ and C20. Today, the new clade candidate may include *V*. *astriarenae* sp. nov. C7^T^ and C20, and *V*. *agarivorans*, and the whole genome sequence of *V*. *agarivorans* that are currently in progress is expected to revealed more. The study will allows direct comparison of both species and describes the new clade more thoroughly. For now, the name Agarivorans is proposed for the new clade.

### Genomic coherence—demonstrates a stable taxonomic framework for the novel strains

A group of novel bacterial species is defined as having >5% mol G+C difference of the genomic DNA, <70% DDH similarity (both experimental and *in silico*), <96% AAI [47] and ANI [6, 43] against closely related species. The pre-determined cut-off values for each parameter is generally in a good correlation to the boundary of 70% DDH similarity [43]. Such genomic coherence provides a stable taxonomic framework for species identification and is expected to acquire a certain degree of phenotypic consistency. The DNA G+C content of the novel strains are 46.4 and 46.1% for C7^T^ and C20 respectively. The mol percentages fall within the range of genus vibrio i.e. 46 to 52% [48] and support our initial phenotypic, and *pyrH* and 16S rRNA phylogeny data (Figs 1 and 2). In spite of all the debates over the reliability of the parameters used and/or the circumscription threshold [2–3,6], our result is in agreement with Tindall et al. [43] as they pointed out that the DNA G+C content is still a useful parameter. Apparently, the HPLC-based DNA G+C content of C7^T^ and C20 has placed them into the genus *Vibrio*.

DNA-DNA hybridization results showed that two strains of *V*. *astriarenae* sp. nov. C7^T^ and C20 were conspecific when genomic DNA of *V*. *astriarenae* sp. nov. C7^T^ was used as probe. *pyrH* phylogeny data (Fig 2) had showed a close relation of both strains to *V*. *agarivorans* in support to their similar agarolytic characters. However, experimental DDH using *V*. *agarivorans* CECT 5085^T^ as a probe showed only 17.3% and 25.7% DNA-DNA relatedness against C7^T^ and C20, respectively. The DNA-DNA relatedness of strains C7^T^ and C20 were 8.4% and 7.1%, respectively, against *V*. *hangzhouensis* JCM 15146^T^ as a probe. Previous 16S rRNA phylogeny data (Fig 1) also suggested a close relationship of novel bacteria to Mediterranei and Orientalis clades. Later, experimental DDH against *V*. *brasiliensis* LMG 20546^T^ as a probe revealed only 9.7% and 12.1% relatedness for C7^T^ and C20, respectively.

Available draft genome sequence of *V*. *astriarenae* sp. nov. C7^T^, *V*. *maritimus* C210, *V*. *variabilis* C206, *V*. *brasiliensis* LMG 20546^T^, *V*. *mediterranei* AK1^T^, *V*. *orientalis* ATCC 33934^T^, *V*. *sinaloensis* DSM 21326 and *V*. *tubiashii* ATCC 19109^T^ has allowed simultaneous *in silico* analyses to provide a more rigid argument over their novelty. *In silico* DDH (%) values (Formula 2, recommended) of C7^T^ against *V*. *maritimus* C210, *V*. *variabilis* C206 and *V*. *mediterranei* AK1^T^ were 31.6 ± 2.5%, 31.9 ± 2.5% and 30.5 ± 2.5%, respectively. Furthermore, *in silico* DDH (%) values of C7^T^ against *V*. *brasiliensis* LMG 20546^T^, *V*. *orientalis* ATCC 33934^T^, *V*. *sinaloensis* DSM 21326 and *V*. *tubiashii* ATCC 19109^T^ (representatives of Orientalis clade) were 31.4±2.5%, 30.9±2.5%, 31.4±2.5% and 32.0±2.5%, respectively. The values have further discerned C7^T^ from other of its immediate group.

In addition to that, separate analysis on the average amino acid identity (AAI) revealed the distant relatedness of C7^T^ against all seven vibrios. The AAI values of C7^T^ against *V*. *maritimus* C210, *V*. *variabilis* C206, *V*. *mediterranei* AK1^T^, *V*. *brasiliensis* LMG 20546^T^, *V*. *orientalis* ATCC 33934^T^, *V*. *sinaloensis* DSM 21326 and *V*. *tubiashii* ATCC 19109^T^ were 71.4, 71.8, 70.3, 73.4, 73.7, 73.5 and 73.5%, respectively. Similarly, C7^T^ was also found to have low average nucleotide identity (ANI) against *V*. *maritimus* C210, *V*. *variabilis* C206, *V*. *mediterranei* AK1^T^, *V*. *brasiliensis* LMG 20546^T^, *V*. *orientalis* ATCC 33934^T^, *V*. *sinaloensis* DSM 21326 and *V*. *tubiashii* ATCC 19109^T^ at 84.6, 84.8, 83.6, 84.7, 84.6, 84.7 and 84.8%, respectively. ANI is claimed to be the most acknowledged parameter used for microbial classification [6] and both AAI and ANI values described herein are below the threshold for species circumscription and we can consider that C7^T^ belongs to a new species [32,47].

### Phenotypic coherence—validates a common physiological, structural and ecological characters among organisms of the same taxon

In microbial taxonomy classification, a new species within a genus can be distinguished by a set of specific phenotypic tests. The tests may include specific cultural characteristics, enzymes production and metabolism of specific organic compounds. Thus, the organisms of the same taxon do exhibit some degree of phenotypic coherence. Upon inoculation of seawater specimens collected from the coral reef off Iriomote-Ishigaki islands, two isolates with pronounced agarolytic activity were recovered from TCBS agar. The cells are rods and appear to be motile with polar flagella. Bacterial colonies are circular with the entire margin producing shallow craters when cultured in ZoBell 2216E agar. These bacteria required salt for its growth, facultative anaerobes and were catalase and gelatinase positive. They produced lipase and DNase and ferment D-glucose, D-galactose, trehalose, D-mannitol and N-acetylglucosamine. Specific biochemical and physiological features of *V*. *astriarenae* sp. nov. are shown in Table 1. It is noted that, the phenotypic profiles of both *V*. *astriarenae* sp. nov. C7^T^ and C20 were almost identical except for some variables in oxidase reaction, nitrate reduction, acid production from D-glucose and assimilation of melibiose and lactose. The type strains were also compared to *V*. *agarivorans* CECT 5085^T^ and the profiles revealed some differences in carbon source use. Novel strains were capable of utilizing D-mannose, D-gluconate, trehalose and DL-malate as sole carbon and energy sources while *V*. *agarivorans* did not (Table 1). Phenotypic and biochemical features of the reference strains including *V*. *hangzhouensis* JCM 15146^T^ and *V*. *agarivorans* CECT 5085^T^ are also presented in S2 Table as way of comparison to the novel strains [49–50]. The sole carbon source assimilation for both reference strains was tested in the basal medium of Baumann and Baumann using Biotype^R^-100 strips (bioMerieux) containing 99 pure carbon sources including a negative control. The growth of the bacteria tested was recorded after incubation at selected temperature and time interval. The test allows direct comparison to our strains as it also requires the cells to grow and assimilate the carbon sources rather than a mere colour changes displayed by other identification kits. The results are depicted in Table 1. Other references were also included and presented in S2 Table, but be aware that the sole carbon assimilation tests for *V*. *maritimus* LMG 25439^T^, *V*. *variabilis* LMG 25438^T^ and *V*. *brasiliensis* LMG 20546^T^ were performed using the API ZYM, API 20E (bioMerieux) and Biolog GN2 metabolic fingerprinting kits [48,51].

**Table 1:** Useful phenotypic and genotypic characteristics for distinguishing *Vibrio astriarenae* sp. nov. and *Vibrio agarivorans*.

Characteristics		*V*. *astriarenae* C7^T^	*V*. *agarivorans* CECT 5085^T^
DNA G+C content (mol %)		46.4	44.8
DDH against DNA from (%)	*V*. *astriarenae* sp. nov. C7^T^	100	41.9
	*V*. *agarivorans* CECT 5085^T^	17.3	100
Growth in/at			
	0.5% (w/v) NaCl	−	+
	37°C	−	+
Production of			
	Amylase	+	−
	Gelatinase	+	−
Nitrate reduction		−	+
Utilization of			
	D-Mannose	+	−
	D-Gluconate	+	−
	Trehalose	+	−
	DL-Malate	+	−

Data for utilization of organic compounds by *V*. *agarivorans* CECT 5085^T^ were obtained from Macián et al. (2004).

Note that, the phenotypes displayed are the result of gene expression leading to some metabolic pathways or function in the cells. In this current phase of next-generation sequencing (NGS) and bioinformatics advances, the phenotypes of a novel strain can be predicted by *in silico* phenotyping. Using the protocol proposed in Amaral et al. [5] the gene coding for the major diagnostic phenotypes of the novel vibrio species were detected in the genome sequences of *V*. *astriarenae* sp. nov. C7^T^, suggesting that diagnostic phenotypic features can be retrieved directly from genome sequences [5]. Thirteen phenotypes were extracted namely ornithine and arginine decarboxylation, indole, acetoin production and fermentation of arabinose, sucrose, galactose, cellobiose, D-mannitol, trehalose, D-sorbitol, myo-inositol and D-mannose. A prototype vibriophenotyping program by Amaral et al. [5] allows an automated comparative analysis of C7^T^ with 35 other *Vibrionaceae*. Some representatives of Mediterranei (*V*. *mediterranei* and *V*. *maritimus*) and Orientalis clade (*V*. *orientalis* and *V*. *tubiashii*) were also included in the analyses. The results are correlated to experimental phenotypic characters in which 9 of 13 phenotypes tested were found to be negative and utilization of trehalose was found to be positive in both methods (S3 Table). On the other hand, utilization of galactose, mannitol and cellobiose was found to be positive in laboratory methods but negative in the *in silico* phenotyping.

Despite the potential of *in silico* phenotyping, phenotypic identification in general, either by classic experiment or *in silico* still suffers much lower resolution and has a more limited scope compared to genome-based identification [5]. Phenotypic observation of the sister species including *V*. *cholerae* and *V*. *harveyi* for instance, reveals a relatively high similarity (>65%) against its genomic counterpart with ≤25% *in silico* DDH indicating their individuality—different species [5]. Regarding the data presented herein, three *in vitro* phenotypes were found to be incongruent in their *in silico* tests. The incongruence between the two tests may result in either negative *in vitro* but positive *in silico* or vice versa. The former case has already been explained in Amaral et al. [5,52] in which the expression of genes *in vitro* may be arrested by some mutations and/or the absence of a global regulator responsible for the phenotype. However, in the latter case (positive *in vitro*, negative *in silico*) observed in fermentation of galactose, mannitol and cellobiose herein, the possible explanation may include an irregular manner or alternative routes of the metabolism of these compounds as described in the assimilation of fructose in *E*. *coli* [52–53]. Also, low Pearson correlation of only 0.68 between phenotypic and genotypic similarity may in part explain the discrepancies [5].

Nevertheless, we propose a proper manual examination should be performed when using the automated phenotyping method particularly on every gene involved in the contradicted phenotype (the phenotypes that are different in *in vitro* and *in silico* experiment). The amino acid sequence of the genes can be used as queries for BLAST searches and re-verify their identity. For instance, theoretically, a total of six genes should be present for the positive assimilation of galactose. Taking a close look at each gene involved, only α-D-galactose 1-phosphate permease was claimed not to be found in *V*. *astriarenae* sp. nov. C7^T^ with only 28.5% similarity. However, BLAST searches of the gene reveals up to 88% identity to symporter YagG of *Vibrio* spp. and with 96% query coverage, it shows 85% similarity to the putative permease of *Photobacterium profundum*. The similarity is high and these proteins also belong to the sugar porter family of a major facilitator superfamily (MFS). Positive assimilation of galactose *in vitro* by C7^T^ may be due to this similarity. Still, thorough investigation is needed before any other conclusion is made. In support to the prototype program developed for *Vibrionaceae* [5], frequent usage of the program on many *Vibrio* species will further validate and possibly draw its own circumscription value in determining a new species. Thus, based on *in silico* phenotyping alone, strain C7^T^ is found to be closely related to *V*. *alginolyticus* supporting the above grouping method which grouped them together with *Vibrio* species. This experiment with combination of phylogenetic analyses (Figs 1, 2 and 3) showed that strains C7^T^ and C20 should be recognized not only as a new species but also as a new clade. The name *Vibrio astriarenae* sp. nov. is proposed for strains C7^T^ and C20 indicating the islands from where they were originated.

## Conclusion

In conclusion, all polyphasic taxonomic data combined with the genome based taxonomy now has confirmed the justification of our proposal of *V*. *astriarenae* sp. nov. as a new species of vibrio. Both *pyrH* and 16S rRNA gene sequence phylogenies, the most fundamental and strong selective measures, had grouped the strains into the genus *Vibrio* and distinguished them from any known *Vibrio* spp. Despite having similar agarolytic activity, the strains differed from *V*. *agarivorans* CECT 5085^T^ and its other closest relatives. Further genomic analyses including DNA G+C content, DNA-DNA hybridization (DDH), genomic sequencing, AAI, ANI and *in silico* DDH of the type strain C7^T^ provided a clear result that the *V*. *astriarenae* sp. nov. is the newest member of *Vibrionaceae*. Later, MLSA of eight housekeeping genes for both C7^T^ and C20 placed them into a new clade within the family *Vibrionaceae* together with *V*. *agarivorans* CECT 5085^T^. The clade is named Agarivorans and further research and the sequencing of whole genome of *V*. *agarivorans* CECT 5085^T^ and C20 is currently in progress. Comparative data on the basis of phenotypic characteristics also supports their novelty (Table 1 and S2 Table) and simultaneously places them into the genus *Vibrio*. Thirteen phenotypic features of *V*. *astriarenae* sp. nov. C7^T^ can be retrieved directly from its genome sequence using an automated vibriophenotyping program

Approaching genomic taxonomy, many debates [2–3,6] over the reliability and relevance of DDH and polyphasic taxonomy together as the “golden standard” for microbial taxonomy arose. Our article in brief depicts the real current situation of such evolution in microbial taxonomy by describing a new species of *Vibrionaceae* which we named *V*. *astriarenae* sp. nov. C7^T^. In our stance, we agree that once genomic taxonomy is realized, it may provide a much better description of new species. Indeed, with the current available genomic data in many public and private databases, genomic technology and many bioinformatics tools, we anticipate genomic taxonomy may complement or eventually replace polyphasic taxonomy in the near future. However, we are aware that such progress should not be rushed or otherwise the outcome may be counterproductive. We also believe, in the next few years, microbiological taxonomy will witness a very progressive movement towards a new coherent prokaryote species concept and most probably with vibrios as an excellent test model [46].

### Description of *Vibrio astriarenae* sp. nov.

*V*. *astriarenae*. (as.tri.a.re'nae. L. n. astrum, star; L. n. arena, sand. N.L. gen. n. astriarenae, from ‘starry sand’. This refers to the isolation source of the sample site, Taketomi Island which is known for its starry sand due to the remains of foraminifera. The shells of these single-celled protists formed star shaped grains of sand along the beaches of Taketomi Island).

Gram-negative, facultatively anaerobic, motile with polar flagella rod isolated from seawater specimen collected from coral reef of Taketomi Island (24°20.5260' N; 124°05.6443' E). Cells are rod shaped, with rounded ends, and are 0.7 to 1.1 μm in diameter and 2.4 to 3.1 μm long when the organism is grown in ZoBell 2216E agar. Polar flagella is observed when the organism is cultivated on solidified media and/or in liquid media. Colonies on ZoBell 2216E agar medium are non-pigmented, circular and smooth with entire edge. Sodium ion is essential for growth. Growth occurs at NaCl concentrations of 1.0 to 3.0% with optimum growth and apparent agarolytic activity at 3.0% NaCl. No growth is detected at 5.0% NaCl and beyond for both strains. Both strains grew at pH 5 to 10, optimally in pH 7.5. No growth was observed at pH >10. The bacterium is a mesophilic chemoorganotroph which grows at temperatures between 20 and 30°C. No growth occurs at 15, 37 and 40°C. The type strain is negative for oxidase and positive for catalase, production of amylase, lipase, agarase, gelatinase and DNase; and assimilation of d-glucose, d-xylose, d-mannose, d-galactose, trehalose, cellobiose, melibiose, lactose, d-gluconate, dl-malate, d-mannitol and *N*-acetylglucosamine. The bacterium is negative for gas production from glucose, acid production from glucose, nitrate reduction, acetoin, caseinase and urease production, lysine decarboxylase, arginine dehydrolase, ornithine decarboxylase, indole, luminescence, pigmentation; assimilation of sucrose, d-glucuronate, propionate, citrate, pyruvate, d-sorbitol, γ-aminobutyrate, putrescine, l-tyrosine and l-arabinose. The G+C content of the DNA from the type strain is 46.3 mol%. The strain C7^T^ was deposited in the Japan Collection of Microorganisms, Collection of Aquacultural Important Microorganisms, Mexico and BCCM/LMG Bacteria Collection, Belgium under respective accession numbers, JCM 19233^T^, CAIM 1900^T^ and LMG 28701^T^.

## Supporting Information

S1 Table List of reference strains used for the phylogenetic tree based on 16S rRNA gene sequence as shown in Fig 1. (DOCX)

S2 Table Useful phenotypic characters for distinguishing *Vibrio astriarenae* sp. nov. with their closely related species. (DOCX)

S3 Table Comparative *in silico* phenotypic characters of *V*. *astriarenae* sp. nov. C7^T^ with other *Vibrionaceae*. (DOCX)

S4 Table Accession number of the reference strains used for the concatenated tree based on eight housekeeping gene sequences as shown in Fig 3. (DOCX)

S1 Dataset The sequences of eight housekeeping gene of *Vibrio astriarenae* sp. nov. C7^T^ used for MLSA. (DOCX)
