# Supplementary material for: Advanced Microbial Taxonomy Combined with Genome-Based-Approaches Reveals that Vibrio astriarenae sp. nov., an Agarolytic Marine Bacterium, Forms a New Clade in Vibrionaceae
Source: PLoS One. 2015 Aug 27;10(8):e0136279. doi: 10.1371/journal.pone.0136279 (PMC4551953; doi:10.1371/journal.pone.0136279)
Supplement: S1 Table — (DOCX) [file pone.0136279.s001.docx]

Table S1: List of reference strains used for the phylogenetic tree based on 16S rRNA gene sequence as shown in figure 1.

| Bacterial strains | Accession number | Citation^*^ |
| --- | --- | --- |
| *Aliivibrio finisterrensis* CECT 7228^T^ | EU541604 | Beaz-Hidalgo et al. 2010 |
| *A. fischeri* NCIMB 1281^T^ | X70640 | Beijerinck 1889 |
| *A. logei* ATCC 29985^T^ | AJ437616 | Harwood et al. 1980 |
| *A. salmonicida* NCIMB 2262^T^ | X70643 | Egidius et al. 1986 |
| *A. sifiae* KTCT 22535^T^ | AB464964 | Yoshizawa et al. 2010 |
| *A. wodanis* LMG 24053^T^ | AJ132227 | Lunder et al. 2000 |
| *Enterovibrio calviensis* DSM 14347^T^ | AF118021 | Denner et al. 2002 |
| *E. coralii* LMG 22228^T^ | AJ842343 | Thompson et al. 2005 |
| *E. nigricans* CAIM 661^T^ | AM942722 | Pascual et al. 2009 |
| *E. norvegicus* LMG 19839^T^ | AJ316208 | Thompson et al. 2002 |
| *Escherichia coli* K-12 | NC_010473 | Durfee et al. 2008 |
| *Grimontia hollisae* ATCC 33564^T^ | X74707 | Hickman et al. 1982 |
| *G. marina* KCTC 22666^T^ | FJ943235 | Choi et al. 2013 |
| *Photobacterium aestuarii* JCM 18592^T^ | JF751050 | Jeon et al. 2014 |
| *P. angustum* ATCC 25915^T^ | X74685 | Reichelt et al. 1979 |
| *P. aphoticum* CECT 7614 | FN796493 | Lucena et al. 2011 |
| *P. aplysiae* JCM 12948^T^ | AY781193 | Seo et al. 2005 |
| *P. aquae* JCM 18480^T^ | JQ948040 | Liu et al., 2014 |
| *P. aquimaris* KCTC 22356^T^ | AB428873 | Yoshizawa et al. 2009 |
| *P. damselae* subsp. *damselae* ATCC 33539^T^ | X74700 | Love et al. 1982 |
| *P. frigidiphilum* JCM 12947^T^ | AY538749 | Seo et al. 2005 |
| *P. gaetbulicola* CCUG 58399^T^ | GQ260188 | Kim et al. 2010 |
| *P. ganghwense* JCM 12487^T^ | AY960847 | Park et al. 2006 |
| *P. halotolerans* LMG 22194^T^ | AY551089 | Rivas et al. 2006 |
| *P. iliopiscarium* ATCC 51760 | AY643710 | Onarheim et al. 1995 |
| *P. indicum* ATCC 19614^T^ | AB016982 | Johnson and Weisrock 1969 |
| *P. jeanii* CAIM 1817^T^ | GU065210 | Chimetto et al. 2010 |
| *P. kishitanii* LMG 23890^T^ | AY341439 | Ast et al. 2007 |
| *P. leiognathi* ATCC 25521^T^ | X74686 | Boisvert et al. 1967 |
| *P. lipolyticum* DSM 16190^T^ | AY554009 | Yoon et al. 2005 |
| *P. lutimaris* JCM 13586^T^ | NR_043902 | Jung et al. 2007 |
| *P. phosphoreum* ATCC 11040^T^ | X74687 | Beijerinck 1889 |
| *P. profundum* JCM 10084^T^ | D21226 | Nogi et al. 1998 |
| *P. rosenbergii* LMG 22223^T^ | AJ842344 | Thompson et al. 2005 |
| *P. swingsii* CAIM 1393^T^ | GQ386822 | Gomez-Gil et al. 2011 |
| *Salinivibrio costicola* subsp. *alcaliphilus* DSM 16359^T^ | AJ640132 | Romano et al. 2005 |
| *S. costicola* subsp. *costicola* ATCC 35508^T^ | X74699 | Smith 1938 |
| *S. costicola* subsp. *vallismortis* DSM 8285^T^ | AF057016 | Huang et al. 2000 |
| *S. proteolyticus* DSM 19052^T^ | DQ092443 | Amoozegar et al. 2008 |
| *S. sharmensis* DSM 18182^T^ | AM279734 | Romano et al. 2011 |
| *S. siamensis* JCM 14472^T^ | AB285018 | Chamroensaksri et al. 2009 |
| *Vibrio aerogenes* LMG 19650^T^ | AF124055 | Shieh et al. 2000 |
| *V. aestivus* CAIM 1861^T^ | HE613734 | Lucena et al. 2013 |
| *V. aestuarianus* ATCC 35048^T^ | X74689 | Tison and Seidler 1983 |
| *V. agarivorans* CECT 5085^T^ | AJ310647 | Macian et al. 2001 |
| *V. albensis* ATCC 14547^T^ | EF032499 | Lehmann and Neumann 1896 |
| *V. alfacsensis* CAIM 1831^T^ | JF316656 | Gomez-Gil et al. 2012 |
| *V. alginolyticus* ATCC 17749^T^ | X74690 | Miyamoto et al. 1961 |
| *V. anguillarum* ATCC 19264^T^ | X16895 | Bergeman 1909 |
| *V. areninigrae* JCM 14949^T^ | EU143360 | Chang et al. 2008 |
| *V. artabrorum* LMG 23865^T^ | EF599164 | Diéguez et al. 2011 |
| *V. atlanticus* LMG 24300^T^ | EF599163 | Diéguez et al. 2011 |
| *V. atypicus* LMG 24781^T^ | FJ009624 | Wang et al. 2010 |
| *V. azureus* KCTC 22352^T^ | AB428897 | Yoshizawa et al. 2009 |
| *V. brasiliensis* LMG 20546^T^ | AJ316172 | Thompson et al. 2001 |
| *V. breoganii* LMG 23858^T^ | EF599161 | Beaz Hidalgo et al. 2009 |
| *V. campbelli* ATCC 25920^T^ | X56575 | Baumann et al. 1971 |
| *V. caribbeanicus* DSM 23640^T^ | GU223601 | Hoffman et al 2012 |
| *V. casei* DSM 22364^T^ | FJ968722 | Bleicher et al. 2010 |
| *V. celticus* LMG 23850^T^ | EF599162 | Beaz Hidalgo et al. 2010 |
| *V. chagasii* CAIM 431^T^ | AJ316199 | Thompson et al. 2003 |
| *V. cholerae* ATCC 14035^T^ | X74695 | Pacini 1854 |
| *V. cincinnatiensis* ATCC 35912^T^ | X74698 | Brayton et al. 1986 |
| *V. comitans* LMG 23416^T^ | DQ922915 | Sawabe et al. 2007 |
| *V. coralliilyticus* LMG 20984^T^ | AJ440005 | Ben-Haim et al. 2003 |
| *V. cortegadensis* LMG 27474^T^ | HF955040 | Lasa et al. 2014 |
| *V. crassostreae* CAIM 1405^T^ | EF094887 | Faury et al. 2004 |
| *V. cyclitrophicus*  LMG 21359^T^ | AM162656 | Hedlund and Staley 2001 |
| *V. diabolicus* CNCM I-1629^T^ | X99762 | Raguénès et al. 1997 |
| *V. diazotrophicus* ATCC 33466^T^ | X74701 | Guerinot et al. 1982 |
| *V. ezurae* JCM 21522^T^ | AY426980 | Sawabe et al. 2005 |
| *V. fluvialis* ATCC 33809^T^ | X76335 | Lee et al. 1981 |
| *V. fortis* LMG 21557^T^ | AJ514916 | Thompson et al. 2003 |
| *V. furnisii* ATCC 35016^T^ | X76336 | Brenner et al. 1984 |
| *V. gallaecicus* CECT 7244^T^ | EU541605 | Beaz Hidalgo et al. 2009 |
| *V. gallicus* CIP 107863^T^ | AY257972 | Sawabe et al. 2004 |
| *V. gazogenes* ATCC 29988^T^ | X74705 | Baumann et al. 1981 |
| *V. gigantis* LMG 22741^T^ | EF094888 | Le Roux et al. 2005 |
| *V. halioticoli* IAM14596^T^ | AB000390 | Sawabe et al. 1998 |
| *V. hangzhouensis* JCM 15146^T^ | EU082035 | Xu et al. 2009 |
| *V. harveyi* ATCC 35084^T^ | X74693 | Baumann et al. 1981 |
| *V. hemicentroti* DSM 26178^T^ | JX204734 | Kim et al. 2013 |
| *V. hepatarius* LMG 20362^T^ | AJ345063 | Thompson et al. 2003 |
| *V. hippocampi* LMG 25354^T^ | FN421434 | Balcazar et al. 2010 |
| *V. hispanicus* CAIM 525^T^ | AY254042S1 | Gomez-Gil et al. 2004 |
| *V. ichthyoenteri* LMG 19664^T^ | AJ421445 | Ishimaru et al. 1996 |
| *V. inusitatus* LMG 23434^T^ | DQ922920 | Sawabe et al. 2007 |
| *V. jasicida* JCM 16453^T^ | AB562589 | Yoshizawa et al. 2012 |
| *V. kanaloae* LMG 20539^T^ | NR_114804 | Thompson et al. 2001 |
| *V. lentus* CECT 5110^T^ | AJ278881 | Macian et al. 2001 |
| *V. litoralis* DSM 17657^T^ | DQ097523 | Nam et al. 2007 |
| *V. mangrovi* DSM 19641^T^ | EU144014 | Rameshkumar et al. 2011 |
| *V. maritimus* CAIM 1455^T^ | GU929925 | Chimetto et al. 2011 |
| *V. marisflavi* CECT 7928^T^ | FJ847833 | Wang et al. 2011 |
| *V. mediterranei* CIP 103203^T^ | X74710 | Pujalte and Garay 1986 |
| *V. metoecus* LMG 27764^T^ | KJ647312 | Kirchberger et al. 2014 |
| *V. metschnikovii* JCM 21189^T^ | X74711 | Gamaleia 1888 |
| *V. mimicus* ATCC 33653^T^ | X74713 | Davis et al. 1982 |
| *V. mytili* ATCC 51288^T^ | X99761 | Pujalte et al. 1993 |
| *V. natriegens* ATCC 14048^T^ | X74714 | Payne et al. 1961 |
| *V. navarrensis* CIP 103381^T^ | X74715 | Urdaci et al. 1991 |
| *V. neonatus* JCM 21521^T^ | AY426979 | Sawabe et al. 2005 |
| *V. neptunius* LMG 20536^T^ | AJ316171 | Thompson et al. 2003 |
| *V. nereis* ATCC 25917^T^ | X74716 | Harwood et al. 1980 |
| *V. nigripulchritudo* ATCC 27043^T^ | X74717 | Baumann et al. 1971 |
| *V. ordalii* ATCC 33509^T^ | X74718 | Schiewe et al. 1982 |
| *V. orientalis* ATCC 33934^T^ | X74719 | Yang et al. 1983 |
| *V. ostreicida* CECT 7398^T^ | AJ296159 | Prado et al. 2014 |
| *V. owensii* LMG 25443^T^ | GU018180 | Cano-Gomez 2010 |
| *V. pacinii* LMG 19999^T^ | AJ316194 | Gomez-Gil et al. 2003 |
| *V. parahaemolyticus* ATCC 17802^T^ | X74720 | Sakazaki et al. 1963 |
| *V. pectenicida* CAIM 594^T^ | Y13830 | Lambert et al. 1998 |
| *V. pelagius* CECT 4202^T^ | AJ293802 | Baumann et al. 1971 |
| *V. penaeicida* DSM 14398^T^ | AJ421444 | Ishimaru et al. 1995 |
| *V. plantisponsor* MSSRF60^T^ | GQ352641 | Rameshkumar et al. 2011 |
| *V. pomeroyi* LMG 20537^T^ | AJ491290 | Thompson et al. 2003 |
| *V. ponticus* CECT 5869^T^ | AJ630103 | Macian et al. 2004 |
| *V. porteresiae* DSM 19223^T^ | EF488079 | Rameshkumar et al. 2008 |
| *V. proteolyticus* ATCC 15338^T^ | X74723 | Merkel et al. 1964 |
| *V. quintilis* CAIM 1863^T^ | HE613736 | Lucena et al. 2013 |
| *V. rarus* LMG 23674^T^ | DQ914239 | Sawabe et al. 2007 |
| *V. rhizosphaerae* MSSRF3^T^ | DQ847123 | Rameshkumar and Nair 2007 |
| *V. rotiferianus* LMG 21460^T^ | AJ316187 | Gomez-Gil et al. 2003 |
| *V. ruber* JCM 11486^T^ | AF462458 | Shieh et al. 2003 |
| *V. rumoiensis* FERM P-14531^T^ | AB013297 | Yumoto et al. 1999 |
| *V. sagamiensis* KCTC 22354^T^ | AB428909 | Yoshizawa et al. 2010 |
| *V. scophthalmi* LMG 19158^T^ | U46579 | Cerdà-Cuéllar et al. 1997 |
| *V. sinaloensis* CAIM 797^T^ | DQ451211 | Gomez-gil et al. 2008 |
| *V. splendidus* ATCC 33125^T^ | X74724 | Beijerinck 1900 |
| *V. stylophorae* LMG 25357^T^ | GQ281380 | Sheu et al. 2011 |
| *V. superstes* CAIM 904^T^ | AY155585 | Hayashi et al. 2003 |
| *V. tapetis* CECT 4600^T^ | Y08430 | Borrego et al. 1996 |
| *V. tasmaniensis* LMG 20012^T^ | AJ316192 | Thompson et al. 2003 |
| *V. toranzoniae* CECT 7225^T^ | HE978310 | Lasa et al. 2013 |
| *V. tritonius* AM2^T‡^ |  | Sawabe et al. 2013 |
| *V. tubiashii* ATCC 19109^T^ | X74725 | Hada et al. 1984 |
| *V. variabilis* LMG 25438^T^ | GU929924 | Chimetto et al. 2011 |
| *V. vulnificus* ATCC 27562^T^ | X76333 | Reichelt et al. 1979 |
| *V. xiamenensis* DSM 22851^T^ | GQ397859 | Gao et al. 2012 |
| *V. xuii* LMG 21346^T^ | AJ316181 | Thompson et al. 2003 |

*Citations were from [1] and NCBI database

‡Data not yet published
