# Supplementary material for: Advanced Microbial Taxonomy Combined with Genome-Based-Approaches Reveals that Vibrio astriarenae sp. nov., an Agarolytic Marine Bacterium, Forms a New Clade in Vibrionaceae
Source: PLoS One. 2015 Aug 27;10(8):e0136279. doi: 10.1371/journal.pone.0136279 (PMC4551953; doi:10.1371/journal.pone.0136279)
Supplement: S2 Table — (DOCX) [file pone.0136279.s002.docx]

**Table S2.** Useful phenotypic characters for distinguishing *Vibrio astriarenae* sp. nov. with their closely related species

| **Characteristics** |  | **1** | **2** | **3** | **4** | **5** | **6** |
| --- | --- | --- | --- | --- | --- | --- | --- |
| Pigmentation |  | － | － | － | － | b＋ | bg+ |
| Growth in/at |  |  |  |  |  |  |  |
|  | 8% (w/v) NaCl | － | － | － | w+ | － | － |
|  | 0.5% (w/v) NaCl | － | ＋ | ＋ | ＋ | ＋ | ＋ |
|  | 15^o^C | － | － | － | ＋ | ＋ | ＋ |
|  | 37^o^C | － | ＋ | ＋ | ＋ | ＋ | ＋ |
|  | 40^o^C | － | － | － | － | ＋ | ＋ |
| Oxidase |  | － | ＋ | ＋ | ＋ | ＋ | ＋ |
| Hydrolysis of |  |  |  |  |  |  |  |
|  | Starch | ＋ | ＋ | － | NA | NA | NA |
|  | Gelatin | ＋ | ＋ | － | ＋ | ＋ | ＋ |
|  | Tween 80 | ＋ | － | w+ | ＋ | ＋ | － |
| Arginine dehydrolase |  | － | － | － | ＋ | ＋ | ＋ |
| Indole production |  | － | ＋ | － | ＋ | ＋ | ＋ |
| Acid from |  |  |  |  |  |  |  |
|  | D-Glucose/D-Mannitol/Maltose/Sucrose | － | ＋ | － | ＋ | ＋ | ＋ |
| Utilization of |  |  |  |  |  |  |  |
|  | D-Mannose | ＋ | ＋ | － | ＋ | ＋ | ＋ |
|  | Sucrose | － | ＋ | － | ＋ | ＋ | ＋ |
|  | D-Gluconate | ＋ | ＋ | － | － | － | NA |
|  | Sorbitol | － | － | － | － | － | ＋ |
|  | Melibiose | ＋ | NA | ＋ | － | － | － |
|  | Lactose | ＋ | ＋ | ＋ | － | － | NA |
|  | D-Glucuronate | － | NA | － | ＋ | ＋ | NA |
|  | Trehalose | ＋ | － | － | ＋ | ＋ | NA |
|  | γ-Aminobutyrate | － | NA | － | － | ＋ | ＋ |
|  | Pyruvate | － | ＋ | － | ＋ | m+ | m+ |
|  | D-Glucosamine/*N*-Acetylglucosamine | ＋ | NA | ＋ | ＋ | ＋ | － |
|  | meso-Erythritol/D-Xylose | ＋ | － | ＋ | － | ＋ | NA |
|  | Citrate | － | ＋ | － | － | － | NA |

Species described herein are: 1, *V. astriarenae* sp. nov. (n = 2); 2, *V. hangzhouensis* JCM 15146^T^; 3, *V. agarivorans* CECT 5085^T^; 4, *V. maritimus* LMG 25439^T^; 5, *V. variabilis* LMG 25438^T^; *V. brasiliensis* LMG 20546^T^. Phenotypic data for reference species were obtained from Xu et al. (2009); Macián et al. (2004); Chimetto et al. (2011); and Thompson et al. (2003). +, strains tested positive; –, strains tested negative; F, fermentative response; b+, colonies may turn black after some time under limited light condition; bg+, beige colonies on TSA; m+, positive for fermentation of methyl pyruvate; w+, weakly positive; NA, data not available.
