# Supplementary material for: Advanced Microbial Taxonomy Combined with Genome-Based-Approaches Reveals that Vibrio astriarenae sp. nov., an Agarolytic Marine Bacterium, Forms a New Clade in Vibrionaceae
Source: PLoS One. 2015 Aug 27;10(8):e0136279. doi: 10.1371/journal.pone.0136279 (PMC4551953; doi:10.1371/journal.pone.0136279)
Supplement: S3 Table — (DOCX) [file pone.0136279.s003.docx]

| **Table S3.** Comparative *in silico* phenotypic characters of *V. astriarenae* C7^T^ with other *Vibrionaceae*. | | | | | | | | | | | | | |
| --- | --- | --- | --- | --- | --- | --- | --- | --- | --- | --- | --- | --- | --- |
|  | Production of: | | | | Utilization of: | | | | | | | | |
|  | Acetoin | Ornithine decarboxylase | Arginine dehydrolase | Indole | Sucrose | Galactose | Cellobiose | D-Mannitol | Trehalose | D-Sorbitol | myo-Inositol | D-Mannose | L-Arabinose |
| 1 | + | v | - | v | + | v | + | + | + | - | - | + | - |
| 2 | + | + | - | + | + | v | - | + | v | - | - | v | - |
| 3 | - | - | - | - | + | v | + | - | + | - | + | + | + |
| 4 | + | - | + | - | - | + | + | - | v | - | - | + | - |
| 5 | - | - | + | v | + | + | V | - | + | - | - | v | + |
| 6 | - | - | + | - | + | v | - | + | + | - | - | + | + |
| 7 | - | - | - | + | + | + | - | + | - | - | - | + | + |
| 8 | + | - | + | - | v | v | - | + | + | v | v | + | - |
| 9 | - | + | - | + | - | + | - | + | + | - | - | - | - |
| 10 | - | + | - | + | - | + | - | + | + | - | - | + | + |
| 11 | - | + | - | + | v | + | + | + | + | - | - | + | - |
| 12 | - | - | + | + | - | + | + | + | + | - | - | + | - |
| 13 | + | - | + | v | + | v | v | + | + | + | - | + | v |
| 14 | - | - | - | v | - | - | + | - | + | - | - | + | - |
| 15 | v | - | v | - | v | v | v | - | v | - | - | v | - |
| 16 | - | - | + | + | - | + | + | - | + | - | - | v | + |
| 17 | - | - | - | - | - | + | + | - | - | - | - | + | - |
| 18 | v | - | - | - | + | v | + | + | + | v | - | + | + |
| 19 | - | + | - | + | + | + | + | + | + | - | - | + | - |
| 20 | - | v | - | - | + | + | + | + | v | - | - | + | - |
| 21 | - | - | + | + | + | + | + | + | + | + | - | + | - |
| 22 | - | - | - | - | v | + | - | - | - | - | - | + | - |
| 23 | - | - | - | - | + | v | v | + | + | - | - | v | + |
| 24 | - | - | + | v | + | - | - | + | + | - | - | + | - |
| 25 | - | - | - | + | - | v | + | - | + | - | - | v | - |
| 26 | - | - | - | - | + | - | - | v | - | - | - | + | - |
| 27 | - | - | v | + | - | - | + | v | + | - | - | + | - |
| 28 | - | - | - | - | - | v | v | v | + | + | - | v | - |
| 29 | - | - | - | + | v | v | - | v | + | - | - | + | - |
| 30 | - | - | + | + | + | - | + | + | + | - | - | + | - |
| 31 | - | - | - | - | + | - | - | + | + | - | - | + | - |
| 32 | - | - | v | + | - | - | + | v | + | - | - | + | - |
| 33 | - | - | v | + | v | - | + | v | + | - | - | - | - |
| 34 | - | - | + | + | + | v | v | + | + | - | - | + | - |
| 35 | - | - | - | - | - | + | + | v | + | - | - | v | - |
| 36 | - | - | - | - | - | - | - | - | - | + | - | - | - |

Species used in vibriophenotyping analysis are: 1, *V. alginolyticus*; 2, *V. cholera*; 3, *V. cincinnatiensis*; 4, *P. damsela*; 5, *V. fluvialis*; 6, *V. furnissii*; 7, *G. hollisae*; 8, *V. metschnikovii*; 9, *V. mimicus*; 10, *V. parahaemolyticus*; 11, *V. vulnificus*; 12, *V. aestuarianus*; 13, *V. anguillarum;* 14, *V. campbellii*; 15, *S. costicola*; 16, *V. diazotrophicus*; 17, *A. fischeri*; 18, *V. gazogenes*; 19, *V. harveyi*; 20, *A. logei*; 21, *V. mediterranei*; 22, *V. maritimus*; 23, *V. natriegens*; 24, *V. nereis*; 25, *V. nigripulchritudo*; 26, *V. ordalli*; 27, *V. orientalis*; 28, *V. pelagius* biogroup 1; 29, *V. pelagius* biogroup 2; 30, *V. proteolyticus*; 31, *A. salmonicida*; 32, *V. splendidus* biogroup 1; 33, *V. splendidus* biogroup 2; 34, *V. tubiashii*; 35, *V. vulnificus* biogroup 2; 36, *V. astriarenae* C7^T^
