# Supplementary material for: Advanced Microbial Taxonomy Combined with Genome-Based-Approaches Reveals that Vibrio astriarenae sp. nov., an Agarolytic Marine Bacterium, Forms a New Clade in Vibrionaceae
Source: PLoS One. 2015 Aug 27;10(8):e0136279. doi: 10.1371/journal.pone.0136279 (PMC4551953; doi:10.1371/journal.pone.0136279)
Supplement: S4 Table — (DOCX) [file pone.0136279.s004.docx]

**Table S4.** Accession number of the reference strains used for the concatenated tree based on eight housekeeping gene sequences as shown in Figure 3.

| Bacterial species | Strain | *ftsZ* | *gapA* | *mreB* | *topA* | *gyrB* | *pyrH* | *recA* | *rpoA* |
| --- | --- | --- | --- | --- | --- | --- | --- | --- | --- |
| *Vibrio aerogenes* | LMG 19650^T^ | KF697253 | KF697261 | KF697278 | KF697313 | KF697270 | KF697286 | KF697295 | KF697304 |
| *V. agarivorans* | CECT 5085^T^ = LMG 21449^T^ = DSM 13756^T^ | KT339396 | FJ436364 | KT339394 | KT339395 | FJ436362 | FJ463225 | KT281478 | KT281479 |
| *V. alginolyticus* | LMG 4409^T^ | EF027344 | DQ907274 | DQ907405 | DQ907472 | AB298202 | GU266285 | AJ842373 | AJ842558 |
| *V. astriarenae* sp*. nov.* | C7^T^  = CAIM 1900^T^ = LMG 28701^T^ | BBMQ01000001-336 (WGS data)* | | | | | | | |
| *V. astriarenae* sp*. nov.* | C20 | KT281480 | KT281481 | KT281483 | KT281486 | KT281482 | KT281484 | KT281487 | KT281485 |
| *V. azureus* | NBRC 104587^T^ | AB428903 | AB428907 | AB428905 | HE655452 | AB428899 | AB428901 | FR669656 | AB465316 |
| *V. litoralis* | DSM 17657^T^ | AUFZ01000001 (WGS data) | | | | | | | |
| *V. rumoiensis* | FERM P-14531^T^ = ST16^T^ | DQ907374 | DQ907307 | DQ907446 | DQ907516 | AB298245 | NZ_AJYK01000090.1 | AJ842503 | AJ842690 |
| *V. tapetis* | LMG 19706^T^ = CECT 4600^T^ | DQ907379 | DQ907311 | DQ907451 | DQ907520 | AB298249 | HE795189 | HE795219 | AJ842730 |
| *V. nigripulchritudo* | LMG 3896^T^ | EF027347 | DQ907297 | DQ907437 | DQ907505 | AB298236 | GU266290 | AJ842480 | AJ842667 |
| *V. proteolyticus* | LMG 3772^T^ = NBRC 13287^T^ | EF114210 | DQ907305 | DQ907444 | DQ907514 | AB298261 | NZ_BATJ01000010 | AJ842499 | AJ842686 |
| *V. gigantis* | LMG 22741^T^ | DQ481629 | DQ481617 | DQ979358 | DQ481655 | AB298219 | EU871951 | EU541593 | EU541573 |
| *V. pomeroyi* | LMG 20537^T^ | DQ481634 | DQ481621 | DQ481646 | DQ481660 | AB298242 | EU871960 | AJ842497 | AJ842684 |
| *V. lentus* | CAIM 454 = R-3884 | DQ914234 | DQ481620 | DQ914236 | DQ914237 | AB298226 | NA | AJ842453 | AJ842640 |
| *V. splendidus* | LMG 19031^T^ | DQ481635 | DQ481622 | DQ481647 | DQ481662 | AB298248 | KF899231 | AJ842511 | AJ842725 |
| *V. kanaloae* | LMG 20539^T^ | DQ481631 | DQ481619 | DQ481643 | DQ481657 | AB298225 | FN908851 | AJ842450 | AJ842637 |
| *V. cyclitrophicus* | LMG21359^T^ | DQ481625 | DQ481613 | DQ481639 | DQ481651 | AB298211 | EU871958 | AJ842405 | AJ842592 |
| *V. chagasii* | LMG 21353^T^ | DQ996590 | DQ481611 | DQ481637 | DQ481649 | AB298206 | EU118252 | AJ842385 | AJ842572 |
| *V. fortis* | LMG 21557^T^ = CECT 8657^T^ | DQ907346 | DQ907282 | DQ907417 | DQ907484 | AB298216 | LN713958 | AJ842422 | AJ842609 |
| *V. pelagius* | ATCC 25916^T^ = CECT 4202^T^ | DQ907369 | DQ907302 | DQ907442 | DQ907511 | AB298241 | LN713960 | AJ580872 | AJ842682 |
| *V. cholerae* | ATCC 39315 | NC0_02505 (WGS data) | | | | | | | |
| *V. mimicus* | LMG 7896^T^ = ATCC 33653^T^ | DQ907357 | DQ907292 | DQ907430 | DQ907498 | AB298230 | EU118242 | EF643485 | EF643486 |
| *V. ‘parilis’* | RC586 | ADBD01000006 (WGS data) | | | | | | | |
| *V. navarrensis* | LMG 15976^T^ = 1397-6^T^ | DQ907360 | DQ907295 | DQ907433 | DQ907501 | AB298233 | KJ807123 | AJ842474 | KJ807163 |
| *V. vulnificus* | LMG13545^T^ = ATCC 27562^T^ | DQ907382 | DQ907313 | DQ907454 | DQ907522 | AB298252 | GQ382226 | GQ382229 | GQ382243 |
| *V. maritimus* | R40493 | GU929927 | KF666681 | GU929931 | GU929939 | KF666700 | GU929933 | GU929935 | GU929937 |
| *V. variabilis* | LMG 25438^T^ = CAIM 1454 = R-40492^T^ | GU929926 | KF666686 | GU929930 | GU929938 | GU929928 | GU929932 | GU929934 | GU929936 |
| *V. mediterranei* | AK1 = LMG 19703 | DQ907377 | DQ907310 | DQ907449 | DQ907496 | NZ_ABCH01000001 | GU266289 | AJ842507 | AJ842695 |
| *V. mediterranei* | LMG11258^T^ | DQ907356 | DQ907290 | DQ907428 | DQ907495 | AB298228 | GU266288 | AJ842459 | AJ842644 |
| *V. owensii* | CAIM 1816 = R40496 | AB609124 | AB609125 | GU078686 | GU078704 | GU078680 | GU078692 | GU078693 | GU078697 |
| *V. harveyi* | LMG4044^T^ = NCIMB1280^T^ = ATCC 14126^T^ | DQ907350 | DQ449616 | DQ907422 | DQ907488 | AB298221 | FM202541 | DQ648369 | KC954196 |
| *V. campbellii* | ATCC BAA1116 | NC_009783 (WGS data) | | | | | | | |
| *V. campbellii* | LMG 11216^T^ | DQ907337 | DQ449614 | DQ907408 | DQ907475 | AB298205 | EF596641 | AJ842377 | AJ842564 |
| *V. rotiferianus* | LMG 21460^T^ | DQ907372 | DQ449619 | DQ907445 | DQ907515 | AB298244 | EF596722 | AJ842501 | AJ842688 |
| *V. tasmaniensis* | LMG 20012^T^ | DQ481636 | DQ481623 | DQ481648 | DQ481661 | AB298250 | EU871961 | AJ842515 | AJ842731 |
| *V. natriegens* | LMG 10935^T^ = CECT 526^T^ | DQ907359 | DQ907294 | DQ907432 | DQ907500 | AB298232 | FM202573 | FM204826 | AJ842658 |
| *V. parahaemolyticus* | LMG 2850^T^ | DQ907367 | DQ449618 | DQ907440 | DQ907509 | AB298239 | GU266286 | AJ842490 | AJ842677 |
| *V. mytili* | LMG 19157^T^ | DQ907358 | DQ907293 | DQ907431 | DQ907499 | AB298231 | GU266287 | HQ455540 | AJ842657 |
| *V. gazogenes* | ATCC 29988^T^ | KF697255 | KF697264 | KF697280 | KF697314 | KF697272 | KF697288 | KF697297 | KF697306 |
| *V. ruber* | LMG 23124^T^ | KF697259 | KF697268 | KF697284 | KF697319 | KF697276 | KF697292 | KF697301 | KF697310 |
| *V. rhizosphaerae* | MSSRF 3^T^ | KF697258 | KF697267 | KF697283 | KF697318 | KF697275 | KF697291 | KF697300 | KF697309 |
| *V. ‘tritonius’* | AM2^T^ | GU951702 | GU969215 | GU969223 | GU969227 | GU969219 | KF697293 | KF697302 | KF697311 |
| *V. porteresiae* | MSSRF 30^T^ | KF697257 | KF697266 | KF697282 | KF697317 | KF697274 | KF697290 | KF697299 | KF697308 |
| *V. diazotrophicus* | LMG7893^T^ = CECT 627^T^ | DQ907342 | DQ907280 | DQ907413 | DQ907480 | AB298212 | HE805632 | AJ842411 | AJ842598 |
| *V. hispanicus* | LMG 13240^T^ | DQ907353 | DQ907286 | DQ907425 | DQ907492 | AB298223 | NA | AJ842445 | AJ842632 |
| *V. fluvialis* | LMG 7894^T^ | DQ907345 | DQ907281 | DQ907416 | DQ907483 | AB298215 | JN426808 | AJ842419 | AJ842606 |
| *V. furnissii* | LMG 7910^T^ = CAIM 518^T^ | EF027345 | DQ907283 | DQ907418 | DQ907485 | AB298217 | JF316672 | AJ842427 | AJ842614 |
| *V. anguillarum* | LMG 4437^T^ | DQ907334 | DQ907275 | DQ907406 | DQ907471 | AB298203 | NA | AJ580852 | AJ842561 |
| *V. ordalii* | LMG 13544^T^ = ATCC 33509^T^ | DQ907364 | DQ907298 | DQ907438 | DQ907506 | AB298237 | NZ_AEZC01000192 | AJ842482 | AJ842669 |
| *V. aestuarianus* | LMG 7909^T^ | DQ907331 | DQ907271 | DQ907402 | DQ907469 | AB298200 | NA | AJ842369 | AJ842554 |
| *V. cincinatiensis* | LMG 7891^T^ | DQ907340 | DQ907278 | DQ907411 | DQ907479 | AB298208 | NA | AJ580853 | AJ842582 |
| *V. metschnikovii* | LMG 11664^T^ | EF027346 | DQ907291 | DQ907429 | DQ907497 | AB298229 | NZ_ACZO01000006 | | AJ842650 |
| *V. ponticus* | DSM 16217 | DQ907371 | DQ907304 | DQ907443 | DQ907513 | AB298243 | JF316676 | JF316685 | JQ308806 |
| *V. scophthalmi* | LMG 19158^T^ | DQ907376 | DQ907309 | DQ907448 | DQ907518 | AB298247 | HM771376 | HM771381 | HM771386 |
| *V. ichthyoenteri* | LMG 19664^T^ = ATCC 700023^T^ | DQ907354 | DQ907287 | DQ907426 | DQ907493 | AB298224 | HM771375 | HM771380 | HM771385 |
| *V. hepatarius* | LMG 20362^T^ | DQ907352 | DQ907285 | DQ907424 | DQ907491 | AB298222 | JF316674 | AJ842444 | AJ842631 |
| *V. orientalis* | LMG 7897^T^ = ATCC 33934^T^ | DQ907365 | DQ907299 | DQ907439 | DQ907507 | AB298238 | EU118243 | EU130528 | AJ842672 |
| *V. tubiashii* | LMG 10936^T^ = ATCC 19109^T^ | DQ907381 | DQ907312 | DQ907453 | DQ907521 | AB298251 | JF316670 | AJ842518 | AJ842734 |
| *V. brasiliensis* | LMG 20546^T^ | DQ907335 | DQ449619 | DQ907407 | DQ907473 | AB298204 | HM771374 | AJ842376 | HM771384 |
| *V. sinaloensis* | DSM21326 | AEVT01000067 (WGS data) | | | | | | | |
| *V. caribbeanicus* | ATCC BAA2122 | AEIU01000122 (WGS data) | | | | | | | |
| *V. corallilyticus* | LMG 20984^T^ | DQ907341 | DQ907279 | DQ907412 | EF114213 | AB298210 | GU266292 | AJ842402 | JN039157 |
| *V. neptunius* | LMG 20536^T^ | DQ907361 | DQ907296 | DQ907435 | DQ907503 | AB298234 | GU266291 | AJ842478 | JN039153 |
| *V. pectenicida* | LMG 19642^T^ | DQ907368 | DQ907301 | DQ907441 | DQ907510 | AB298240 | JN039143 | AJ842491 | AJ842678 |
| *V. nereis* | LMG 3895^T^ | DQ907362 | DQ449617 | DQ907436 | DQ907504 | AB298235 | JN968379 | AJ842479 | AJ842666 |
| *V. xuii* | LMG 21346^T^ | DQ907384 | DQ907315 | DQ907456 | DQ907524 | AB298254 | GU266284 | AJ842529 | AJ842742 |
| *V. superstes* | CAIM 904^T^ = LMG 21323^T^ | KF697260 | KF697269 | KF697285 | KF697320 | KF697277 | KF697294 | KF697303 | KF697312 |
| *V. breoganii* | 1C10 | AKXW01000018 (WGS data) | | | | | | | |
| *V. inusitatus* | CAIM1811 | KF666653 | DQ922912 | KF666719 | KF666765 | KF666698 | EU871957 | EU541600 | EU541579 |
| *V. gallicus* | HT2-1^T^ = LMG 21330^T^ | KF697254 | KF697263 | KF697279 | KF697314 | KF697271 | KF697287 | KF697296 | KF697305 |
| *V. ezurae* | JCM 21522^T^ = LMG 19970 ^T^= DSM 17533^T^ | DQ907343 | AY546645 | DQ907414 | DQ907481 | AB298213 | EU871949 | AJ842413 | AJ842600 |
| *V. halioticoli* | IAM 14596^T^ = LMG 18542 | DQ907349 | AY546638 | DQ907421 | DQ907487 | AB298220 | EU871952 | AJ842430 | AJ842617 |
| *V. neonatus* | JCM 21521^T^ = LMG 19973^T^ | KF697256 | KF697265 | KF697281 | KF697316 | KF697273 | KF697289 | KF697298 | KF697307 |
| *V. crassostreae* | LMG 22240^T^ | DQ481624 | DQ481612 | DQ481638 | DQ481650 | AB298209 | EU871948 | EU541594 | EU541574 |
| *Vibrio sp.* | N418 | AFWD01000095 (WGS data) | | | | | | | |
| *Vibrio sp.* | F6 FF238 | AJYW01000195 (WGS data) | | | | | | | |
| *Vibrio sp.* | F10 9ZB36 | AJYQ01000069 (WGS data) | | | | | | | |
| *Vibrio sp.* | LGP32 | NC011753 (WGS data) | | | | | | | |
| *Vibrio sp.* | EX25 | NC013456 (WGS data) | | | | | | | |
| *Vibrio sp.* | EJY3 | NC_016613 (WGS data) | | | | | | | |
| *Grimontia hollisae* | LMG 17719^T^ = CAIM 625^T^ | EF027348 | DQ907317 | DQ907398 | EF114215 | AB298259 | JF739393 | AJ842351 | AJ842535 |
| *Enterovibrio coralii* | LMG 22228^T^ | DQ907329 | DQ907268 | DQ907396 | EF114217 | AB298198 | JF739392 | AJ842347 | AJ842530 |
| *E. norvegicus* | LMG 19839^T^ = CAIM 430^T^ | EF027349 | DQ907269 | DQ907397 | EF114216 | AB298198 | JF739391 | AJ842348 | AJ842531 |
| *Enterovibrio sp.* | 1F-230 | AJYH01000076 (WGS data) | | | | | | | |
| *Enterovibrio sp.* | AK16 | NZ_ANFM01000001 (WGS data) | | | | | | | |
| *Salinivibrio costicola subsp. costicola* | LMG 11651^T^ | EF02735 | DQ907316 | DQ907399 | DQ907468 | AB298255 | ASAI01000001 | AJ842367 | AJ842552 |
| *Photobacterium angustum* | ATCC 25915^T^ = LMG 8455^T^ | DQ907318 | DQ907257 | DQ907385 | DQ907457 | AB298187 | EF380235 | EF415544 | AJ842538 |
| *P. damselae subsp. piscicida* | DI21 | AKYG01000013 (WGS data) | | | | | | | |
| *P. damselae subsp. damselae* | ATCC 33539^T^ = LMG 7892^T^ | DQ907319 | DQ907258 | DQ907386 | DQ907458 | AB298188 | EF380236 | AJ842357 | AJ842541 |
| *P. leiognathi* | NCIMB 2193^T^ = ATCC 25521^T^ | DQ907324 | DQ907263 | DQ907391 | DQ907463 | AB298193 | EF380238 | EF415546 | EF415581 |
| *P. iliopiscarius* | DSM 9896^T^ = ATCC 51760^T^ | DQ907322 | DQ907261 | DQ907389 | DQ907461 | AB298191 | EF380237 | EF380245 | EF380251 |
| *P. lutimaris* | CAIM 1851 | KF666644 | KF666663 | KF666709 | KF666753 | KF666688 | KF666723 | KF666730 | KF666737 |
| *P. phosphoreum* | IAM 14401^T^ = ATCC 11040^T^ | DQ907326 | DQ907265 | DQ907393 | DQ907465 | AB298195 | EF380239 | EF415550 | EF415585 |
| *P. profundum* | SS9 = ATCC BAA-1253 | CR378673 (WGS data) | | | | | | | |
| *Aliivibrio sifiae* | KCTC 22535 = H1-1 | AB464982 | AB464977 | AB464992 | AB464987 | AB464968 | AB464972 | AB464996 | AB465000 |
| *A. fischeri* | LMG 4414^T^ = ATCC 7744^T^ | DQ907344 | AY546637 | DQ907415 | DQ907482 | AB298214 | EF415528 | EU907941 | AJ842604 |
| *A. wodanis* | NCIMB 13582^T^ = ATCC BAA-104^T^ | DQ907383 | DQ907314 | DQ907455 | DQ907523 | AB298253 | EU118246 | EU257781 | EF380250 |
| *A. logei* | LMG 14011 | DQ907355 | DQ907289 | DQ907427 | DQ907494 | AB298227 | KF697321 | AJ842456 | AJ842643 |
| *A. salmonicida* | LFI 1238 | NC_011312 (WGS data) | | | | | | | |
| *Escherichia coli* | K-12 | NC_010473 (WGS data) | | | | | | | |

NA: Not available

* Sequences are presented in Dataset S1
