# Supplementary material for: Advanced Microbial Taxonomy Combined with Genome-Based-Approaches Reveals that Vibrio astriarenae sp. nov., an Agarolytic Marine Bacterium, Forms a New Clade in Vibrionaceae
Source: PLoS One. 2015 Aug 27;10(8):e0136279. doi: 10.1371/journal.pone.0136279 (PMC4551953; doi:10.1371/journal.pone.0136279)
Supplement: S1 Dataset — (DOCX) [file pone.0136279.s005.docx]

**Dataset S1.** The sequences of eight housekeeping gene of *Vibrio astriarenae* sp. *nov* C7^T^ used for MLSA.

>ftsZ_V.astriarenaeC7_ BBMQ01000001-336

ATGTTTGAACCGATGAATGAAATGTCAGACGATGCAGTAATTAAAGTCGTAGGTGTTGGC

GGCGGCGGCGGTAACGCTGTCGAACACATGGTTAGAGAGTCTATCGAAGGCGTACAGTTC

GTTAGTGTTAACACTGATGCACAGGCACTTCGCAAGTCTACAGTAAACAGCGTAATCCAG

ATCGGTGGTGATATGACCAAAGGTTTGGGTGCTGGTGCAAACCCTCAAGTAGGGCGTGAC

GCAGCTCTCGAAGACAGAGAAAGAATTAAAGAAGAGCTAGTAGGCGCCGATATGGTGTTT

ATAGCTGCTGGTATGGGCGGCGGTACAGGTACCGGTGCTGCACCAGTGATTGCTGAAGTG

GCGAAAGAGCTTGGCATTCTTACTGTTGCTGTTGTGACGAAACCATTCAGTTTTGAAGGT

AAAAAGCGTTTGGCGTTTGCCGAGCAGGGTATTGATGAGCTTTCTAAGCACGTTGACTCT

TTGATTACTATTCCAAACGAGAAACTGCTTAAGGTTCTTGGCCGAGGAATCACACTGCTA

GAAGCGTTTGCTAGCGCGAACGATGTTCTAAAAAATGCGGTTCAGGGTATTGCCGAGCTG

ATTACTCGCCAGGTATGA

>gapA_V.astriarenaeC7_BBMQ01000001-336

ATGACTATCAAAGTAGGTATTAATGGTTTTGGCCGTATCGGTCGTTTCGTTTTCCGTGCA

GCACAAGAGCGCGCTGACATCGAAGTAGTAGGTATCAACGACCTAATCGACGTAGATTAC

ATGGCTTACATGCTTAAGTATGACTCAACTCACGGCCGTTTCAACGGTACTGTTGAAGTT

GAAGGCGGTAACCTAATCGTTAACGGTAAAACTGTACGTGTTACAGCTGAGCGTAACCCA

GAAGACCTAAAATGGGACGAAATCGGTGTTGACGTTGTTGCTGAAGCAACGGGTCTTTTC

CTAACTGACGAGACTGCACGTAAGCACATCACTGCTGGTGCGAAGAAAGTTGTTCTAACT

GGTCCTTCAAAAGACGCTACTCCAATGTTCGTTAGCGGCGTAAACTTCGACACTTACGCT

GGTCAAGACATCGTTTCTAACGCTTCTTGTACTACTAACTGTCTAGCGCCTATCGCTAAA

GTTCTTAACGACAAGTTCGGTATCGAGTCTGGTCTAATGACTACAGTTCACGCTACTACA

GCAACTCAAAAAACTGTAGATGGCCCTTCTGCTAAAGACTGGCGCGGTGGCCGTGGTGCT

TCTCAGAACATCATCCCATCTTCAACTGGTGCTGCTAAAGCTGTAGGCGTTGTACTTCCA

GAAGTAAACGGCAAACTAACTGGTATGGCTTTCCGCGTACCAACTGCTAACGTTTCTGTA

GTTGACCTAACAGTTAACCTTAAGAACGGTGCTTCTTACGAAGCTATCTGTGCAGCTATG

AAAGAAGCTTCTGAAGGCGAAATGGCTGGCGTTCTTGGTTACACTGAAGACCAAGTTGTT

TCTCAAGACTTCATCGGTGAAGTTTGCACTTCAGTATTCGATGCTAAAGCTGGTATCGCA

CTTAACGACAAGTTCGTTAAAGTTGTATCTTGGTACGACAACGAAATCGGTTACTCAAAC

AAAGTTCTAGACCTAATCGCTCACGTATCTAAGTAA

>gyrB_V.astriarenaeC7_BBMQ01000001-336

ATGCACCCAGAAGAGAAAGTTTCTGCGGCAGAAGTTATCATGACGGTACTTCACGCTGGT

GGTAAGTTTGATGACAACTCATACAAAGTTTCGGGCGGTCTGCACGGTGTAGGTGTTTCG

GTAGTAAACGCACTGTCTAAGCAAGTCACACTCACGATTCATCGTGGTGGCCAAATTCAT

ACTCAAACCTATCACCATGGTGAGCCTCAAGCGCCACTATCGGTGATTGGCGATACTGAC

AAAACGGGTACAGAAATCCGTTTCTGGCCAAGTGAAGAGACATTCTCAAACACTGAATTC

CATTACGATATCCTAGCTAAGCGTCTACGTGAGCTTTCTTTCCTGAACTCTGGCGTATCG

ATCAAGTTACGTGATGAGCGTGAAGACGATAAAGGTGACCACTTCATGTATGAAGGTGGT

ATCCAAGCGTTCGTAGAGCACTTGAACACCAACAAGACACCAATTATCGAAAAAGTATTC

CACTTTAACGCAGAGCGTGAAGATGGCATTGCTGTAGAAGTCGCAATGCAGTGGAACGAT

GGCTACCAAGAAAATATCTACTGTTTCACCAACAACATCCCTCAGCGTGATGGTGGTACT

CACCTTGCTGGTTTCCGTGCGGCCCTAACTCGTACCCTGAACTCGTTCATGGACAAAGAA

GGTTTCTCCAAGAAAGCGAAAACAGCCACATCAGGTGACGATGCGCGTGAAGGTCTAACG

GCCGTGATCTCGGTTAAAGTGCCAGATCCTAAGTTCTCAAGCCAGACGAAAGATAAGCTC

GTTTCTTCTGAGGTGAAATCAGCGGTTGAATCTGCAATGGGTGAGAAGCTATCTGAGTTC

TTGATTGAGCACCCTGCAGAAGCGAAAACGGTGTGTACTAAGATCATCGATGCAGCGCGT

GCACGTGATGCAGCGCGTAAAGCACGTGAGATGACTCGTCGTAAAGGCGCACTCGACCTT

GCGGGTCTTCCAGGCAAACTTGCTGACTGTCAGGAAAAAGATCCTGCACTGTCTGAACTC

TACATAGTGGAGGGTGACTCGGCAGGCGGCTCCGCAAAACAAGGCCGTAACCGTAAGAAT

CAAGCGATTCTTCCTCTGAAAGGTAAAATCCTGAACGTTGAGAAGGCGCGTTTCGACAAG

ATGCTGTCTTCACAAGAAGTTGCAACGCTAATCACAGCACTTGGCTGTGGTATCGGTCGT

GACGAGTACAACCCAGACAAACTGCGTTACCACAACATCATCATCATGACCGATGCCGAT

GTCGATGGTTCGCACATCCGTACGCTTCTTCTGACCTTCTTCTACCGTCAAATGCCAGAG

CTTATTGAGCGTGGCTACGTGTACATTGCTCAGCCACCACTCTACAAAGTAAAGAAAGGC

AAACAAGAGCAGTACATCAAGGATGAAGATGCAATGAACCAATACCAGGTTTCACTTGCA

CTGGATAACGCAGCACTGCATGTCAATGCTGAGGCGCCAGCACTCGCAGGCGAAGCACTA

GAGTCATTGGTTAAGCAATACAACAAAGGCATGAAGCTGGTGGAGCGCATGAGCCGTCGC

TACCCACAACCACTTGTGCATGAGCTTGTTTACACGCCGCGTCTAACGGCAGAGCAGTGT

CATGATGCCGCTGCTGTTGAAGCTTGGACTAAGACACTCGTAGAGCAGCTAAATGCGAAA

GAAGTGGGTGCAAGCCAGTACAGCTTCGAGATCGAACAGCATGCAGAACTGGGTCTAAAT

CTGCCGAAAATCGTTGTGCGTACACACGGTGTGGTTCATGAGCATGTGCTTTCTGTTGAT

TTCATCAATTCGAAAGAGTACGGCAAGCTCGCTGACCTTTCTGAGGCACTTGATGGTCTT

ATCGAAGAAGGGGCTTACGTGAAGCGTGGTGAGCGCACACTCGCTGTAAGCAGTTTTGTT

GATGCCCTGAACTGGTTGATTAAAGAGTCACGTCGTGGTCTAAGCCTACAGCGCTACAAA

GGTCTAGGTGAGATGAACCCAGATCAGCTATGGGAAACCACCATGGACCCAGATACTCGC

CGTATGATGCAAGTAACGATTGAAGATGCGGTTGGCGCTGACCAGTTGTTTACCACGTTA

ATGGGTGACCAAGTTGAGCCTCGTCGTAACTTCATCGAAGAGAACGCTCTGAAAGTAGCA

AACCTAGACGTATAG

>mreB_V.astriarenaeC7_BBMQ01000001-336

ATGTTCAAAAAACTCCGTGGCATGTTTTCGAACGACCTATCGATCGATTTAGGTACTGCC

AACACTCTGATTTATGTAAAAGGCCAGGGCATTGTCCTTGATGAGCCTTCAGTCGTTGCT

ATCCGTCAGGACAAAGGTCGTGGCGGTAAAACCGTAGCTGCAGTTGGCCATGCCGCGAAA

CAGATGCTAGGTCGTACACCTGGCAACATTTCTGCGATTCGTCCGATGAAAGATGGTGTT

ATTGCCGACTTCTACGTTACTGAAAAAATGCTTCAGCACTTTATCAAGCAAGTGCATGAA

AACAGCTTCCTTAAGCCAAGCCCTCGCGTATTGGTTTGTGTTCCTTGTGGCTCTACACAA

GTTGAGCGCCGTGCAATCCGTGAATCAGCACTCGGTGCAGGTGCTCGTGAGGTTTACCTG

ATTGATGAGCCAATGGCAGCAGCAATCGGTGCTGGCCTGCGCGTATCAGAGCCAACGGGT

TCGATGGTGGTCGATATCGGTGGTGGTACCACTGAAGTTGCCGTTATCTCACTGAATGGT

GTGGTTTACTCTTCATCGGTACGTATCGGTGGTGACCGCTTTGATGAAGCGATCATCAAC

TACGTGCGCCGTAACTACGGCAGCTTGATTGGTGAAGCAACGGCAGAGAAGATCAAACAC

GACATCGGTTCAGCTTACCCAGGTGATGAAGTGCTTGAGATTGAAGTGCGCGGTCGTAAC

CTTGCAGAAGGTGTGCCACGCAGCTTTAGCCTAAACTCAAATGAGATCCTAGAAGCACTG

CAAGAGCCACTAACGGGTATCGTATCAGCTGTCATGGTTGCACTAGAACAGTGTCCACCA

GAGCTGGCATCCGACATCTCTGAAAATGGCATGGTACTGACGGGTGGTGGTGCGCTATTG

CGTGACCTTGACCGCCTAATTGCAGAAGAGACTGGTATCCCTGTTGTGATCGCTGAAGAA

CCTCTAACGTGTGTTGCTCTTGGTGGCGGTAAAGCGCTAGAGATGATCGACATGCACGGT

GGCGATCTGTTCAGCGACGAATAA

>pyrH_V.astriarenaeC7_BBMQ01000001-336

GTGATTTTCTCTGCAGGTACAGGTAACCCGTTCTTCACTACAGATTCAGCAGCGTGTCTA

CGTGGTATTGAAATCGAAGCTGACGTTGTTCTTAAAGCAACAAAAGTTGACGGTGTTTTC

ACGTCAGACCCGGTAGCAAACCCAGATGCTGAACTACTTTCACACCTATCTTATGCTGAA

GTTCTAGATAAAGAGCTAAAAGTAATGGATCTTGCTGCGTTCACTCTTGCTCGTGACCAC

AAAATGCTAATTCGTGTATTCAACATGAATAAGCCTGGTGCACTTCGTCGTGTTGTGATG

GGTGAAGCGGAAGGCACGCTAATTAGTGACGCAGAGTAA

>recA_V.astriarenaeC7_BBMQ01000001-336

ATGGACGAGAACAAACAGAAAGCGCTCGCCGCTGCGCTAGGTCAGATTGAAAAGCAATTC

GGTAAAGGTTCTATTATGCGCCTTGGTGACAACCGCGCAATGGACGTTGAAACGATTTCT

ACTGGCTCACTGTCTTTGGACATCGCACTTGGTGCTGGTGGCCTTCCAATGGGGCGTATC

GTTGAGATCTACGGTCCAGAATCATCGGGTAAAACAACACTGACTCTAGAGCTTATCGCT

GCCGCGCAGAAAGTGGGTAAAACGTGTGCGTTCGTTGATGCTGAGCACGCTCTTGACCCT

ATCTATGCACAAAAGCTAGGCGTTGATATTGATGCGCTGTTGGTTTCTCAACCAGACACG

GGCGAGCAAGCGTTAGAAATCTGTGATGCGTTGGCACGTTCTGGTGCAATCGATGTTCTT

GTTGTCGACTCTGTTGCAGCACTAACTCCAAAAGCTGAAATCGAAGGCGAGATGGGCGAT

AGCCACATGGGTCTTCAAGCACGTATGCTTTCTCAAGCGATGCGTAAGCTAACAGGTAAC

CTAAAGCAGTCTAACTGTATGTGTATCTTCATCAACCAAATCCGTATGAAGATTGGTGTG

ATGTTTGGTAACCCAGAAACAACCACTGGTGGTAATGCACTGAAATTCTACGCTTCTGTT

CGCCTTGATATCCGTCGTACGGGCTCTATCAAAGACGGTGACGAAGTTGTGGGTAACGAA

ACTCGCATCAAAGTGGTTAAGAACAAGATTGCTGCGCCGTTCAAACAAGCTGAAACGCAA

ATTCTCTATGGCCAAGGCTTTAACCGCGAAGGTGAGCTTATTGACCTAGGCGTTAAGCAC

AAGCTGATTGAAAAAGCAGGTGCATGGTACAGCTACAATGGTGACAAGATTGGTCAAGGC

AAAGCCAACGCAGGTAAGTTCCTACGTGAGAACCCTGAAGCGGCTCAAACGATTGATGCT

AAACTGCGTGAAATGCTGCTAACACCAGCACAACCAGAAGCACCTGAAACAGGTGAAATG

CCTCAAGAAGAAGAGCTATAA

>rpoA_V.astriarenaeC7_BBMQ01000001-336

ATGCAGGGTTCTGTAACAGAATTTCTTAAGCCACGTCTTGTTGATATCGAACAAATCAGC

ACGACTCACGCAAAAGTAACTCTTGAGCCATTAGAGCGTGGTTTCGGCCATACTCTTGGT

AATGCACTTCGCCGTATTCTACTATCTTCTATGCCAGGTTGTGCTGTAACAGAAGTAGAG

ATTGAAGGCGTACTACACGAATACAGCACTAAAGAAGGTGTTCAGGAAGATATTCTTGAA

GTTCTTCTTAACCTTAAAGGTCTTGCTGTTCGCGTTGCCGAAGGCAAAGATGAAGTGTTT

ATTACGTTGAACAAATCAGGCTCAGGCCCTGTGGTTGCAGGTGACATCACCCATGATGGT

GATGTAGAGATCGTAAACCCTGAACACGTAATTTGTCACTTAACTGATGACAACGCTGAG

ATCGCTATGCGCATCAAAGTAGAACGTGGTCGTGGTTACGTTCCAGCTTCAGCTCGTATC

CATACTGAAGAAGATGAGCGTCCAATTGGTCGTTTGCTAGTAGACGCGACTTACAGCCCA

GTAGACAAAATTGCCTACGCGGTTGAAGCAGCTCGTGTTGAACAGCGTACCGACTTGGAC

AAGCTTGTTATCGATATGGAAACGAACGGTACTCTTGAGCCTGAGGAAGCAATCCGTCGT

GCAGCAACTATTCTTGCTGAACAATTGGATGCGTTCGTAGATCTTCGTGATGTACGTGTA

CCTGAGGAGAAGGAAGAGAAGCCAGAATTCGATCCGATCCTACTGCGTCCTGTAGACGAT

CTTGAACTAACAGTTCGCTCTGCTAACTGTCTGAAAGCAGAAGCGATTCACTACATCGGT

GATCTTGTACAGCGCACTGAGGTTGAGCTACTTAAAACGCCTAACCTTGGTAAAAATCTC

TTACTGAGATTAAAGACGTACTTGCATCACGTGGTCTTTCTCTGGGCATGCGCTAGAAAA

CTGGCCACCAGCGTCTATCGCTGA

>topA_V.astriarenaeC7_BBMQ01000001-336

GTGCGGAAGAGAAAGCTCGCATCAAAAAGGAAAAAGACCGTAAGTCTCTCATCAAAAAGA

TGGGTATCGACATACAACGGTTGGGAAGCGAATTACCAAATCCTCCCGGGTAAGGAAAAG

GTTGTTTCTGAGCTGCAAAAACTCGCACAAGACGCTGACTACGTTTATCTCGCAACCGAT

TTGGACCGTGAGGGAGAGGCTATCGCTTGGCACCTTCGCGAGATCATCGGCGGTGATGAA

GAGCGATACAAACGCGTTGTGTTTAATGAAATTACAAAAAACGCGATCCAACAGGCATTC

GAACAGCCGGGTGAGCTAAACATTGACGGTGTCAATGCGCAGCAAGCACGTCGTTTTATG

GATCGTGTCGTGGGCTTTATGGTCTCCCCTTTGCTGTGGAAAAAAGTCGCACGAGGTCTA

TCGGCAGGTCGCGTACAGTCGGTTGCAGTGAAGCTGTTGGTTGAGCGTGAGCGCGAAATC

AAAGCCTTCATTCCAGAAGAGTTCTGGGATATTCATGCCAATACGGTAACAAAGGATACC

ACGGACTTTAGACTGCAAGTTGCACAGAAAGATGGTTCGGCTTTCAAACCGGTGAATGAA

GCGGAAACGAAAGCGGCAATGAGCGTGCTTGAGAAAGCAGCTTATGAAGTGTGTAAGCGC

GAAGACCGTCCAACAAGCAGCAAGCCATCAGCGCCATATATCACGTCGACCCTGCAACAA

GCAGCAAGTACCCGCCTAGGTTACGGCGTGAAAAAGACCATGATGCTGGCTCAGCGCCTC

TATGAGGCGGGTTACATCACTTATATGCGTACAGACTCAACTAACCTAAGTAAAGAGGCT

GTAGAGGCTGCTCGTGAGTTTATTGGTAGTGAGTTTGGTGACGCTTACCTTCCTGCAAGC

CCATTGGTTTATGGCAGCAAAGAGGGTGCACAAGAAGCGCACGAAGCGATTCGTCCTTCC

GATGTTGCCGTGAAAGCTGATGACCTACAAGGTATGGAAGCCGATGCACACAAGCTTTAC

GCTCTGATTTGGAATCAATTCGTTGCCTGTCAAATGACACCTGCGAAGTACGATTCAACC

ACAGTGAGTGTGAAAGCGGCAGAGTATACGCTTAAAGCGAAAGGTCGTATTCTTAAGTTT

GATGGTTGGACGCGTGTACAACGTCCACTAGGCAAAAATGAAGACCAAATTCTCCCAGCA

GTACAAGTGGGTGATGTATTGAGCTTGAAAGATCTTGAGCCGAAACAGCACTTCACTAAG

CCACCAGCGCGATTTACCGAGGCTGCCTTGGTTAAAGAGCTGGAGAAACGTGGTATTGGC

CGTCCTTCGACGTATGCATCGATCATCTCCACAATTCAAGATCGTGGCTACGTGAGAGTG

GATCAACGCCGTTTCTACGCAGAGAAAATGGGCGAAATCGTGACCGATCGTCTCGATGAT

AGCTTTAATGATCTCATGAACTACGACTTCACGGCGCGTATGGAAGAGAAGCTCGACCAA

GTGGCTGAAGGTGAAGTGAACTGGAAGAACATGCTGGATAACTTCTTTGGTGATTTCACC

GGTGAGTTAGAAAAAGCTGAGCTCGATGAAGATCACGGCGGTATGAAACCAAACCATATC

GTGATGACAGACATTGAGTGTCCGACATGTTCGCGTCCAATGGGCATTCGCACCGCTTCT

ACGGGTGTATTCTTGGGCTGTTCTGGCTATGCATTACCGCCGAAAGAGCGTTGTAAGACC

ACCATCAACTTGGGTGATGAAGAGGGGATTATCAATGTTCTTGAAGAAGATGTAGAAACT

GCTGCACTGAGAGCGAAAAAGCGTTGTCCAATCTGTGAGACCGCGATGGATGCGTACCTT

ATCGATGATAAGCGCAAGATGCACGTCTGTGGTAACAACCCGAACTGTGATGGTTATGTG

GTTGAACACGGTGAGTTCAAAGTGAAAGGTTACGATGGACCGGTTGTTGAGTGTGACAAG

TGTGGTTCAGACATGGTGCTGAAAAATGGTCGCTTTGGTAAGTACATGGACTGTACCAAT

GAAGATGTAAGAACACGCGTAAGATTCTGA
